# Unconventional aspects of electronic transport in delafossite oxides

**DOI:** 10.1080/14686996.2017.1393633

**Published:** 2017-11-13

**Authors:** Ramzy Daou, Raymond Frésard, Volker Eyert, Sylvie Hébert, Antoine Maignan

**Affiliations:** ^a^ Normandie Univ, ENSICAEN, UNICAEN, CNRS, CRISMAT, Caen, France.; ^b^ Materials Design SARL, Montrouge, France.

**Keywords:** Delafossites, resistivity, thermopower, Nernst effect, electronic structure, anisotropic materials, magnetism, 50 Energy Materials, 105 Low-Dimension (1D/2D) materials, 106 Metallic materials, 206 Energy conversion / transport / storage / recovery, 401 1st principle calculations, 203 Magnetics / Spintronics / Superconductors

## Abstract

The electronic transport properties of the delafossite oxides $\;{\text{ABO}}_{2}$ are usually understood in terms of two well-separated entities, namely the triangular $\;{\text{A}}^{+}$ and ($\;{\text{BO}}_{2}{)}^{-}$ layers. Here, we review several cases among this extensive family of materials where the transport depends on the interlayer coupling and displays unconventional properties. We review the doped thermoelectrics based on $\;{\text{CuRhO}}_{2}$ and $\;{\text{CuCrO}}_{2}$, which show a high-temperature recovery of Fermi-liquid transport exponents, as well as the highly anisotropic metals $\;{\text{PdCoO}}_{2}$, $\;{\text{PtCoO}}_{2}$, and $\;{\text{PdCrO}}_{2}$, where the sheer simplicity of the Fermi surface leads to unconventional transport. We present some of the theoretical tools that have been used to investigate these transport properties and review what can and cannot be learned from the extensive set of electronic structure calculations that have been performed.

## Graphical Abstract

**Figure:**
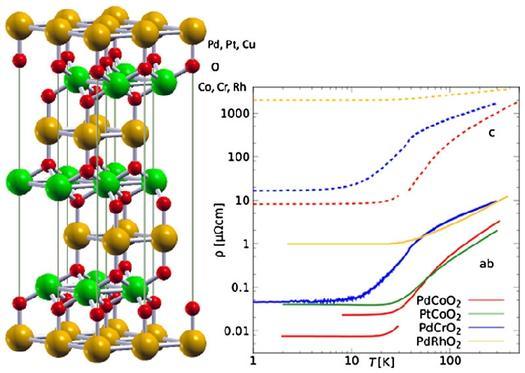

## 1. Introduction

Transition metal oxides attract a lot of attention due to a great variety of physical phenomena, most of which go along with the ordering of some microscopic degrees of freedom as a function of e.g. temperature, pressure, or doping [1]. Prominent examples are the striking metal–insulator transitions in vanadium sesquioxide [2–5], high-$T_{c}$ superconductivity in the cuprates, or the colossal magnetoresistance observed in the manganates [6–9]. Cobaltates have aroused much interest due to the occurrence of different spin states [10–12]. In addition, they are promising materials for thermoelectric applications [13,14].

Known since 1873, when Friedel discovered the mineral ${\mathrm{CuFeO}}_{2}$ [15], the delafossites ${\mathrm{ABO}}_{2}$ continue to generate strong and ever increasing interest [16–18], especially after Kawazoe et al. showed simultaneous transparency and p-type conductivity [19] in ${\mathrm{CuAlO}}_{2}$. This discovery laid the groundwork for the development of transparent optoelectronic devices. Furthermore, the quasi-two-dimensionality of the lattice and the triangular coordination of atoms give rise to exciting physical properties such as strong anisotropy of the electrical conductivity and magnetic frustration effects.

**Figure 1.**
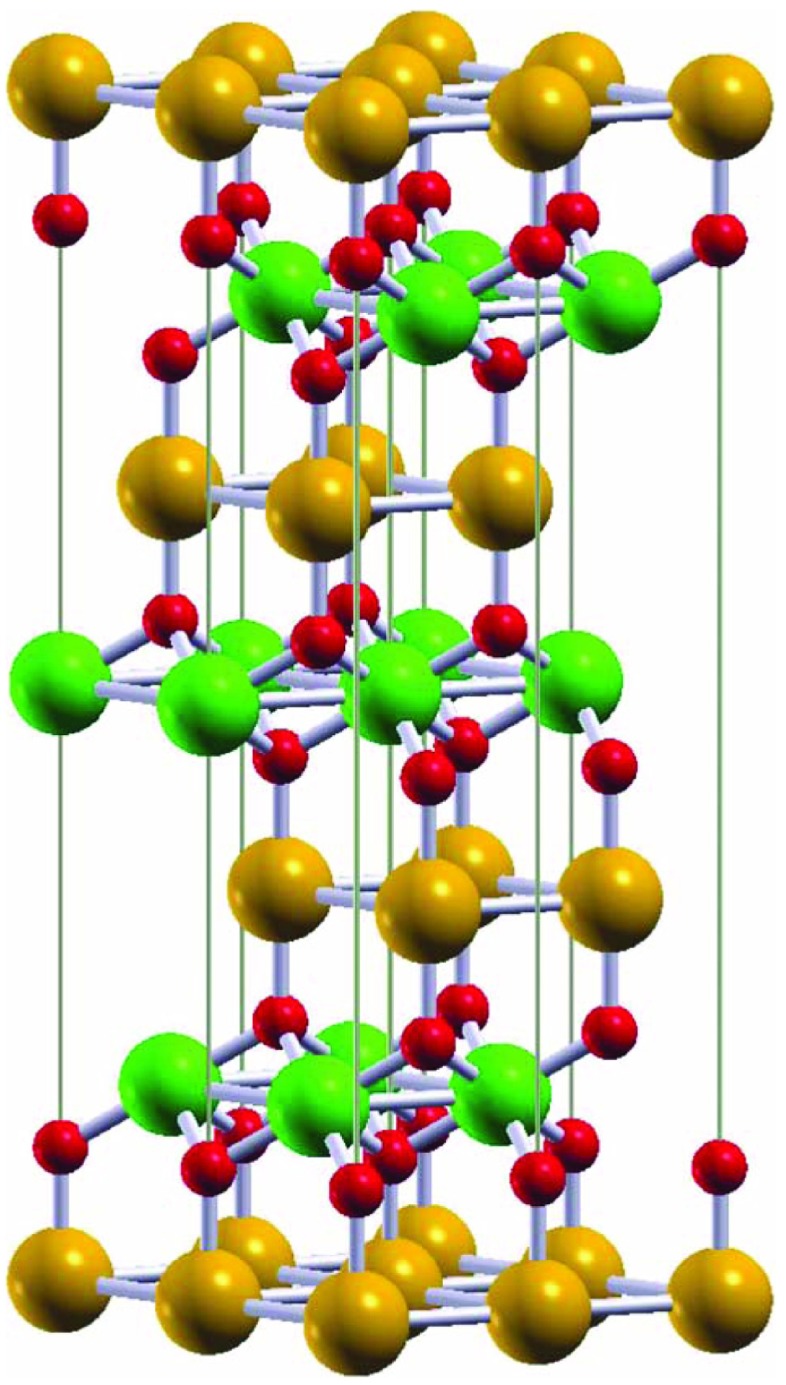
Crystal structure of delafossite $\;{\text{ABO}}_{2}$. A, B, and oxygen atoms are shown as orange, green, and red (light, big dark, and small dark in grayscale) spheres, respectively.

The delafossite structure has the space group $R\overset{¯}{3}\;\text{m}$ and results from a stacking of monoatomic triangular layers, see Figure 1 [16,18]. In particular, the B-atoms are at the centers of edge-sharing distorted oxygen octahedra, which form the characteristic ${\mathrm{BO}}_{2}$ sandwich layers. These trilayers are interlinked by linear O–A–O bonds, resulting in a twofold coordination of the A-atoms. However, the latter have, in addition, six in-plane nearest neighbor A-atoms. For this reason, the structure may be likewise regarded as formed from single A-atom layers, which are intertwined by the octahedral sandwiches. We find this point of view particularly useful when investigating the metallic delafossites. Finally, the oxygen atoms are tetrahedrally coordinated by one A-atom and three B-atoms. Pressure studies on ${\mathrm{PdCoO}}_{2}$ and ${\mathrm{PtCoO}}_{2}$ reveal an increase of the structuralanisotropy on compression indicating the high mechanical stability of both the octahedral sandwich layers and the O–Pd–O (O–Pt–O) dumbbells [20].

Generically, the A and B atoms are mono- and trivalent, respectively. Depending on the chemical composition, a wide variety of behaviors is therefore possible. For instance, if the $A^{+}$ ion is in a $d^{9}$ configuration, metallic conductivity is observed as in the case of ${\mathrm{PdCoO}}_{2}$. If it is in a $d^{10}$ configuration, the degrees of freedom dominating the low-energy physics can be traced back to the B atoms as e.g. in ${\mathrm{CuCrO}}_{2}$, ${\mathrm{AgNiO}}_{2}$ [21,22], and ${\mathrm{AgCrO}}_{2}$ [23,24].

In general, interest in the delafossite-type compounds has focused on the triangular arrangement of the transition-metal atoms and the resulting possible frustration effects, which arise once localized magnetic moments are established. While most of these oxides have been found to be antiferromagnetic semiconductors, other class members like ${\mathrm{PdCrO}}_{2}$, ${\mathrm{PdCoO}}_{2}$, ${\mathrm{PdRhO}}_{2}$, and ${\mathrm{PtCoO}}_{2}$ attracted interest due to their rather high metallic conductivity. In particular, ${\mathrm{PdCoO}}_{2}$ has been shown to possess one of the lowest electric resistivities of normal-state oxides, even lower than that of Pd metal at room temperature [16,17,25]. In fact, with ρ(300K)≃ 2.6μΩcm it is even comparable to pure Au. Yet, the conductivity is strongly anisotropic [16,25]. In particular, the ratio of the resistivities parallel and perpendicular to the *c* axis can be 400 or more in ${\mathrm{PdCoO}}_{2}$ [25,26].

Despite their simple chemical formulae the delafossites may be regarded as prototypical superlattices, where the composition of both the A and B layers can be used to strongly influence the behavior of the whole system. For instance, in ${\mathrm{CuCrO}}_{2}$, the Fermi energy falls into the Cr 3*d* band, but since the Cr layers order magnetically this compound is a magnetic semiconductor. In contrast, as will be shown below, in ${\mathrm{PdCoO}}_{2}$, the Co layers only act a charge reservoirs, and conduction takes place almost exclusively in the Pd layers.

The paper is organized as follows: The experimental results on thermoelectric delafossites $\;{\text{CuCr}}_{1-x}\;{\text{Mg}}_{x}\;{\text{O}}_{2}$ and $\;{\text{CuRh}}_{1-x}\;{\text{Mg}}_{x}\;{\text{O}}_{2}$ are reviewed in Section 2. The properties of metallic delafossites are reviewed in Section 3. In Section 4, we present the theoretical analysis of metallic and thermoelectric delafossites that make up the main body of this review. Conclusions and perspectives are presented in Section 5.

## Thermoelectric delafossites: doped $\;{\text{CuCrO}}_{2}$ and $\;{\text{CuRhO}}_{2}$

2.

**Figure 2.**
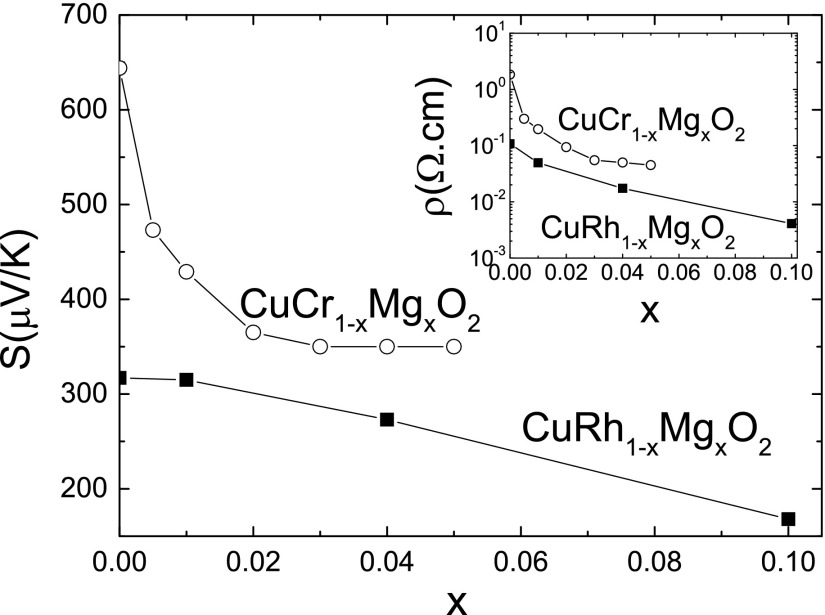
Dependence on Mg content *x* of the thermopower at 300 K of $\;{\text{CuCr}}_{1-x}\;{\text{Mg}}_{x}\;{\text{O}}_{2}$ and $\;{\text{CuRh}}_{1-x}\;{\text{Mg}}_{x}\;{\text{O}}_{2}$. Inset: Dependence on Mg content *x* of the resistivity at 300 K of $\;{\text{CuCr}}_{1-x}\;{\text{Mg}}_{x}\;{\text{O}}_{2}$ and $\;{\text{CuRh}}_{1-x}\;{\text{Mg}}_{x}\;{\text{O}}_{2}$.

Many delafossites behave as semiconductors, with a band gap of order 1 eV, and have been investigated in detail for possible applications in the field of transparent conducting oxides (TCO) [19]. The delafossite family consists in a large number of materials $\;{\text{AMO}}_{2}$, with $\;\text{A}=\;{\text{Ag}}^{+}$, $\;{\text{Cu}}^{+}\ldots$ and M = Al, Ga, Sc, In, Fe, Cr, Rh ... . In this part, we present the results that have motivated our investigation of delafossite electronic structure and their transport properties. We focus on the results related to the doping of $\;{\text{CuCrO}}_{2}$ and $\;{\text{CuRhO}}_{2}$, by substituting $\;{\text{Cr}}^{3+}$ or $\;{\text{Rh}}^{3+}$ by another cation such as $\;{\text{Mg}}^{2+}$, to increase electrical conductivity in order to optimize the thermoelectric properties, as shown in Figure 2. The positive Seebeck coefficient for both pristine delafossites indicates their p-type character and the aliovalent substitution creating more holes in the 3d or 4d bands explains why *S* and ρ decrease with *x*. The properties of metallic delafossites with $\;{\text{d}}^{9}$ A atom ($\;{\text{PdCoO}}_{2}$, $\;{\text{PtCoO}}_{2}$, $\;{\text{PdCrO}}_{2}$ and $\;{\text{PtCrO}}_{2}$) are described in Section 3. In contrast, $\;{\text{CuCrO}}_{2}$ is semiconducting, with a magnetic transition observed at $T_{N}=25$ K toward an antiferromagnetic state. The ratio between $\Theta_{p}$ and $T_{N}$ is very large, close to 7–8 suggesting a large magnetic frustration [27–29]. The possible reduction of the gap by doping has stimulated the investigation of transport properties, and more specifically and more recently, due to the $\;{\text{CdI}}_{2}$ type nature of the $\;{\text{CrO}}_{2}$ layers, isostructural to $\;{\text{CoO}}_{2}$ layers in $\;{\text{Na}}_{x}\;{\text{CoO}}_{2}$, thermoelectric properties have been measured. In 2005, a first report was presented on $\;{\text{CuCr}}_{1-x}\;{\text{Mg}}_{x}\;{\text{O}}_{2}$ [30], probed by specific heat, magnetization and transport measurements up to 300 K. By substituting $\;{\text{Mg}}^{2+}$ on the $\;{\text{Cr}}^{3+}$ site, the Néel temperature $T_{N}$ is kept unchanged as shown by a neutron diffraction study [28], but the Curie–Weiss temperature $\Theta_{p}$ increases from -170 K to -100 K. Considering that $\;{\text{Cr}}^{4+}$ is very difficult to stabilize without high oxygen pressure, the doping effect in this article was interpreted taking into account a mixed valency of $\;{\text{Cu}}^{+}$ / $\;{\text{Cu}}^{2+}$ induced by the $\;{\text{Mg}}^{2+}$ substitution on the $\;{\text{Cr}}^{3+}$ site. Magnetic susceptibility was analyzed taking into account the contribution of both $\;{\text{Cr}}^{3+}$ (S=3/2) and $\;{\text{Cu}}^{2+}$ (S=1/2).

There is a direct impact of the antiferromagnetic ordering on the resistivity curves, with an enhanced magnetoresistance around $T_{N}$ [30]. The transport results (magnetoresistance and resistivity) are consistent with a direct coupling between the doped holes and the $\;{\text{Cr}}^{3+}$ spin. The thermopower remains rather large (still ∼100 μV/K for x=0.02), with values close to the ones of $\;{\text{Na}}_{x}\;{\text{CoO}}_{2}$, but resistivity is actually too high to ensure a large power factor. The authors thus concluded that these delafossites could not be considered for thermoelectric applications.

High-temperature properties were investigated by Ono et al. [31], with electrical resistivity, Seebeck coefficient, and thermal conductivity measured up to 1100 K and for x≤0.05. In this paper, contrary to the interpretation of Okuda et al. [30], the authors argue that a mixed valency of $\;{\text{Cr}}^{3+}$ and $\;{\text{Cr}}^{4+}$ is induced by the $\;{\text{Mg}}^{2+}$ substitution on the Cr site. The Seebeck coefficient dependence on *x* could indeed be interpreted considering the Koshibae and Maekawa formula [32]. The $\;{\text{Cr}}^{3+}$ and $\;{\text{Cr}}^{4+}$ are supposed to be in the high-spin state (S=3/2 and S=1, respectively), and an extra spin entropy term of $69.9\;\mu \;\text{V}\;{\text{K}}^{-1}$ has to be considered. The major contribution to *S* nevertheless originates from the doping by itself, this term being very large due to the very small value of *x*. As the resistivity remains rather large at high *T*, the *ZT* values only increase up to 0.04 for x=0.03. The thermal conductivity is another drawback for large *ZT*, with κ between 4 and $8\;\;\text{W}\;{\text{K}}^{-1}\;{\text{m}}^{-1}$ at 1000 K.Even if $\;{\text{CuCrO}}_{2}$ doped delafossites exhibit modest values of *ZT*, larger *ZT* values have been obtained in $\;{\text{CuRh}}_{0.9}\;{\text{Mg}}_{0.1}\;{\text{O}}_{2}$ and $\;{\text{CuFe}}_{0.99}\;{\text{Ni}}_{0.01}\;{\text{O}}_{2}$ [33,34], with a maximum of 0.15 at 1000 K and 0.14 at 1100 K, respectively. In $\;{\text{CuRh}}_{0.9}\;{\text{Mg}}_{0.1}\;{\text{O}}_{2}$, a smaller thermal conductivity ( ≃ $1\;\;\text{W}\;{\text{K}}^{-1}\;{\text{m}}^{-1}$ at high T), leads to this substantial enhancement of *ZT* with respect to doped $\;{\text{CuCrO}}_{2}$ [33].

Large differences in the *ZT* values are thus observed between $\;{\text{CuCrO}}_{2}$ and these doped $\;{\text{CuRhO}}_{2}$ and $\;{\text{CuFeO}}_{2}$, and the role of $\;{\text{Mg}}^{2+}$ on doping in $\;{\text{CuCrO}}_{2}$ was still unclear. Understanding these differences [30,31] has motivated the reinvestigation of transport properties in $\;{\text{CuCrO}}_{2}$ and $\;{\text{CuRhO}}_{2}$ doped with $\;{\text{Mg}}^{2+}$ [35–37]. X-ray diffraction combined with EDX analysis with transmission electron microscopy has confirmed that the solubility of $\;{\text{Mg}}^{2+}$ is in fact very restricted (x≃0.10 for $\;{\text{CuRhO}}_{2}$ and x≃0.03 for $\;{\text{CuCrO}}_{2}$). In $\;{\text{CuCrO}}_{2}$, the substitution by $\;{\text{Mg}}^{2+}$ rapidly leads to the formation of CuO (observed from x=0.04), and to the formation of the spinel $\;{\text{MgCr}}_{2}\;{\text{O}}_{4}$ as soon as x=0.01 [37]. The evolution of the Seebeck coefficient as a function of *x* shows that doping is induced with the $\;{\text{Mg}}^{2+}$ substitution even above x=0.01 (formation of the spinel $\;{\text{MgCr}}_{2}\;{\text{O}}_{4}$) but is suppressed for x>0.04 as *S* becomes constant. The transport properties have thus been investigated up to 0.04.

The magnetic structure is not strongly affected by the $\;{\text{Mg}}^{2+}$ substitution, as revealed by neutron diffraction [28]. From magnetic susceptibility, the high-spin state for $\;{\text{Cr}}^{3+}$ is confirmed and large Curie–Weiss temperatures (–170 K) compared to a $T_{N}$ of 24 K (weakly affected by Mg substitution) demonstrate the strong magnetic frustration associated to their incommensurate antiferromagnetic structure. In the entire doping range, electrical resistivity exhibits a localized behavior with $\frac{d\rho }{dT}<0$, and large values of ρ close to $10^{-1}$ – $10^{2}\;\Omega \;\;\text{cm}$ at 300 K depending on doping. $\;{\text{Mg}}^{2+}$ substitution leads to a reduction of ρ, see the inset of Figure 2, and for larger $\;{\text{Mg}}^{2+}$ content, ρ can be measured down to low T, with the magnetic transition directly observed at 24 K in the ρ(T) curves. The Seebeck coefficient is positive, and evolves from a localized behavior for x=0 (S∝1/T, with very large values at room temperature close to $650\;\mu \;\text{V}\;{\text{K}}^{-1}$, see Figure 2) to smaller values for x>0, with $\frac{dS}{dT}>0$.

In the antiferromagnetic state, the resistivity and thermopower depend strongly on the magnetic field, as shown by the magnetothermopower and magnetoresistance curves measured up to 9 T. As discussed earlier, the introduction of $\;{\text{Mg}}^{2+}$ could both generate a mixed valency of $\;{\text{Cr}}^{3+}$/$\;{\text{Cr}}^{4+}$ or $\;{\text{Cu}}^{+}$/$\;{\text{Cu}}^{2+}$. The Heikes formula which was used to interpret the *S*(*x*) dependence cannot discriminate between these two different doping origins. However, this existence of magnetoresistance and magnetothermopower supports the $\;{\text{Cr}}^{3+}$/$\;{\text{Cr}}^{4+}$ doping, with the transport being dominated by the Cr–O network rather than the Cu network.

At higher T, for x>0, *S* continuously increases up to 1100 K, while ρ continuously decreases, leading to power factor values close to $2.10^{-4}\;W\;{\text{m}}^{-1}\;{\text{K}}^{-2}$ [38]. This is very close to the values previously reported [31]. More discussion about the *S*(*T*) curves in connection to the band structure can be found in the Section 4.5.

**Figure 3.**
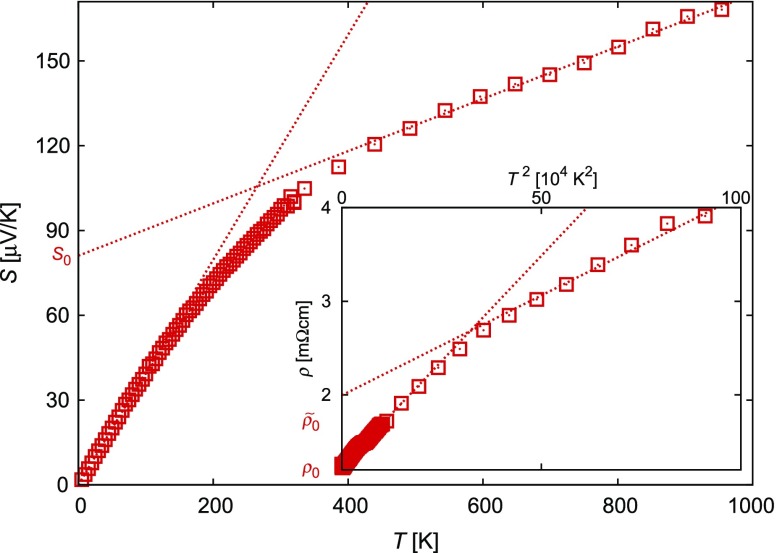
Thermopower *S* and resistivity ρ (inset) of CuRh${}_{0.9}$Mg${}_{0.1}$O${}_{2}$ as functions of the temperature *T* (squares). The linear, respectively, quadratic, regions are highlighted by the dotted lines in order to show the transition from a behavior, which can be explained within a Fermi liquid picture, toward a behavior which is referred to as the one of an apparent Fermi liquid (AFL) and characterized by the additional offsets $S_{0}$ and ${\tilde{\rho }}_{0}$.

In the case of $\;{\text{CuRhO}}_{2}$, $\;{\text{Mg}}^{2+}$ substitution can reach 10%, a value much larger than the one observed in the case of $\;{\text{CuCrO}}_{2}$. $\;{\text{Cu}}_{2}\;{\text{MgO}}_{3}$ appears as an impurity for x>0.10. From band structure calculations, the Cu valency has been assigned to $\;{\text{Cu}}^{+}$, while Rh is $\;{\text{Rh}}^{3+}$ in the low-spin state. With $\;{\text{Mg}}^{2+}$ doping, the semiconducting behavior of $\;{\text{CuRhO}}_{2}$ is gradually replaced by a metallic behavior, for T>100 K in all samples. A minimum of resistivity is observed at $T_{\min}$, even for x=0.10, with $T_{\min}$ decreasing as *x* increases. For large *x*, the resistivity values reach the mΩcm values, typical of the so-called ‘bad metallic’ oxides. Simultaneously, as shown in Figure 2, the thermopower decreases from large values ($325\;\mu \;\text{V}\;{\text{K}}^{-1}$ for x=0), to values close to $170\;\mu \;\text{V}\;{\text{K}}^{-1}$ for x=0.10. It must be emphasized that the Seebeck values for undoped $\;{\text{CuCrO}}_{2}$ are much larger than the ones of $\;{\text{CuRhO}}_{2}$. For the latter, the spin entropy term contributing to only ∼ $70\;\mu \;\text{V}\;{\text{K}}^{-1}$, the larger *S* difference can be ascribed to a higher self-doping in $\;{\text{CuRhO}}_{2}$ naturally resulting from a small off-stoichiometry. The interesting point is that the power factor $\frac{S^{2}}{\rho }$ presents a peculiar *T* dependence, with an almost constant value from 300 to 1000 K for x=0.10, reaching $6.10^{-4}\;W\;{\text{m}}^{-1}\;{\text{K}}^{-2}$, a value typical of the best thermoelectric oxides [37]. This peculiar behavior comes from the $T^{2}$ behavior observed for the electrical resistivity in a large *T* range, associated to an almost *T* linear behavior for *S*. The S∝T and $\rho \propto T^{2}$ behaviors (see Figure 3) have stimulated the development of the Apparent Fermi Liquid model [39].

Another proposed origin of the enhanced Seebeck values in Mg-doped $\;{\text{CuRhO}}_{2}$ is an electronic band structure consisting of a large flat region and a sharply dispersing edge [103], a so-called ’pudding-mold’ model. When the Fermi level is close to the transition between these two regions, the asymmetry can cause an enhanced Seebeck coefficient within the approximations of semi-classical transport.

All the above examples point out the rich variety of properties exhibited by this family of 2D materials. In this ${\mathrm{ABO}}_{2}$ delafossite class of compounds, the pronounced two-dimensionality goes along with the triangular arrangement of transition-metal ions with rather well-localized electrons, which has laid ground for the known variety of extraordinary phenomena. In particular, when the B cation is a paramagnetic 3d metal, though there exists geometric frustration in the antiferromagnetic exchange interactions, the magnetic coupling through the separating A layer is sufficient to allow a setting of a 3D antiferromagnetic ordering as in $\;{\text{CuCrO}}_{2}$ (or $\;{\text{PdCrO}}_{2}$). The competition between the different in- and out-of-plane magnetic interactions is responsible for very different antiferromagnetic structures, collinear in $\;{\text{CuFeO}}_{2}$ or non-collinear in $\;{\text{CuCrO}}_{2}$. In that respect, it is very difficult to predict their spin driven multiferroic properties, ferroelectricity being spontaneous at $T_{N}$ for $\;{\text{CuCrO}}_{2}$ but requiring the application of a magnetic field to be induced in $\;{\text{CuFeO}}_{2}$ [40]. For these antiferromagnetic insulating delafossites with $\;{\text{B}}^{3+}$ cations, doping is necessary to induce more electronic conducting states as the $\;{\text{Mg}}^{2+}$ for $\;{\text{Cr}}^{3+}$ substitution in $\;{\text{CuCrO}}_{2}$, with little effect on $T_{N}$. This behavior contrasts to that of $\;{\text{CuRhO}}_{2}$ with its fully occupied Rh $4d\;t_{2g}$ (S=0) subshell which neither displays strong localization effects nor magnetic order but serves as a possible thermoelectric material as it combines low electrical resistivity and large Seebeck values. Finally, when the A cation is Pd or Pt as in $\;{\text{PdCoO}}_{2}$ and $\;{\text{PtCoO}}_{2}$ (see Section 3), very different electrical properties are observed, the samples behaving as ‘2D metals’ characterized by extreme anisotropy creating a unique situation among all oxides. We point out that these five compounds serve only as paradigmatic examples of the whole class of delafossites and the present survey is far from being exhaustive. Indeed, the richness of phenomena can be further increased by alloying and doping.

## 3. Metallic delafossites

### 3.1. Introduction

The majority of delafossite materials so far synthesized are semiconductors, of particular interest for theirpotential use as p-type transparent oxides. There is, however, a small group of materials that behave quite differently.

When the B site is occupied by Co, Rh, or Cr, the $\;{\text{BO}}_{2}$ layers have an electronic configuration that makes them charged but formally insulating. The octahedral crystal field around the B-site generates the familiar $t_{2g}$ and $e_{g}$ bands, and the $t_{2g}$ bands are either completely filled by low-spin $d^{6}\;\;{\text{Co}}^{3+}$ or $\;{\text{Rh}}^{3+}$, or effectively so by magnetic high-spin $d^{3}$ $\;{\text{Cr}}^{3+}$. The resulting net single negative charge per formula unit requires that the A-site ion carry a single positive charge.

If $\;\text{A}\;=\;\;{\text{Ag}}^{+}$ or $\;{\text{Cu}}^{+}$, the resulting electronic structure is typically gapped, despite the fact that the Ag-Ag or Cu-Cu distance can be shorter than in bulk Ag or Cu. On the other hand, the $d^{9}$ configurations of $\;{\text{Pd}}^{+}$ and $\;{\text{Pt}}^{+}$ produce materials with extremely high, strongly anisotropic conductivity. Conduction in the triangular A-plane is mediated by close overlap between adjacent A-ions, which are separated by distances of 2.830 Å, 2.923 Å, and 3.021 Å  for Pd(Co,Cr,Rh)$\;{\text{O}}_{2}$ [16]. This compares to a nearest neighbor distance of 2.751 Å  in fcc palladium. Likewise in $\;{\text{PtCoO}}_{2}$, the interatomic distance is 2.823 Å[41], compared to 2.775 Å  in the metal. For comparison, the room temperature in-plane resistivities of $\;{\text{PdCoO}}_{2}$ and $\;{\text{PtCoO}}_{2}$ are 2.6μΩcm[43] and 2.1μΩcm[41], much lower than for the fcc metals, which both have around 10.6μΩcm. This dramatic reduction in resistivity for weaker overlap is counterintuitive.

Transport out-of-plane remains metallic in character, even though the separation between consecutive A-planes is as much as 6 Å. Thus, $\;{\text{PdCoO}}_{2}$, $\;{\text{PtCoO}}_{2}$, $\;{\text{PdRhO}}_{2}$[42], and $\;{\text{PdCrO}}_{2}$ are very good metals. $\;{\text{PdCrO}}_{2}$ is additionally notable because of the frustrated antiferromagnetic ordering at 37 K.

De Haas–van Alphen [43] as well as photoemission [44] experiments show that the low-temperature Fermi surface of $\;{\text{PdCoO}}_{2}$ consists of a single electron-like warped hexagonal cylinder, while that of $\;{\text{PdCrO}}_{2}$ resolves into electron- and hole-like bands that arise from magnetic reconstruction of the single sheet [45,46].

An exception to this scheme is $\;{\text{AgNiO}}_{2}$, which also retains metallic character [47]. In this case the $d^{7}\;\;{\text{Ni}}^{3+}$ sets the Fermi energy in the $e_{g}$ band. As the temperature is reduced, orbital degeneracy is lifted by charge transfer between Ni sites and an ordered state arises when 3$\;{\text{Ni}}^{3+}\to \;{\text{Ni}}^{2+}+2\;{\text{Ni}}^{3.5+}$ and metallic conduction is preserved. This contrasts with other $\;{\text{Ni}}^{3+}$ materials where Jahn Teller distortions open a gap between the $e_{g}$ levels and lead to insulating ground states.

Metallic conductivity can also be obtained in many cases by doping, but these are the only stoichiometric delafossites known to produce a metallic ground state comparable to elemental metals. By contrast, the ‘bad metal’ nature of the thermoelectric delafossites discussed in the previous section requires electronic correlations to be taken into account. Below we focus on the transport properties of the clean Pd/Pt materials, where the strong structural anisotropy and simple electronic structure make them textbook cases of quasi-2D metals and give rise to effects rarely seen in the solid state.

### 3.2. Transport properties

#### 3.2.1. Resistivity

$\;{\text{PdCoO}}_{2}$, $\;{\text{PdCrO}}_{2}$, and $\;{\text{PtCoO}}_{2}$ are notable for their very low room temperature in-plane resistivities, ρ(300K)= 2.6μΩcm[43], 10μΩcm[48], and 2.1μΩcm, respectively [41]. This applies to transport confined to the ab-plane. Transport in the inter-plane direction maintains a metallic temperature dependence, although it is typically between 300 and 1000 times more resistive in nature [43,49]. This indicates that there is still some degree of overlap between Pd/Pt planes, perhaps mediated by the $\;{\text{BO}}_{2}$ layers.

**Figure 4.**
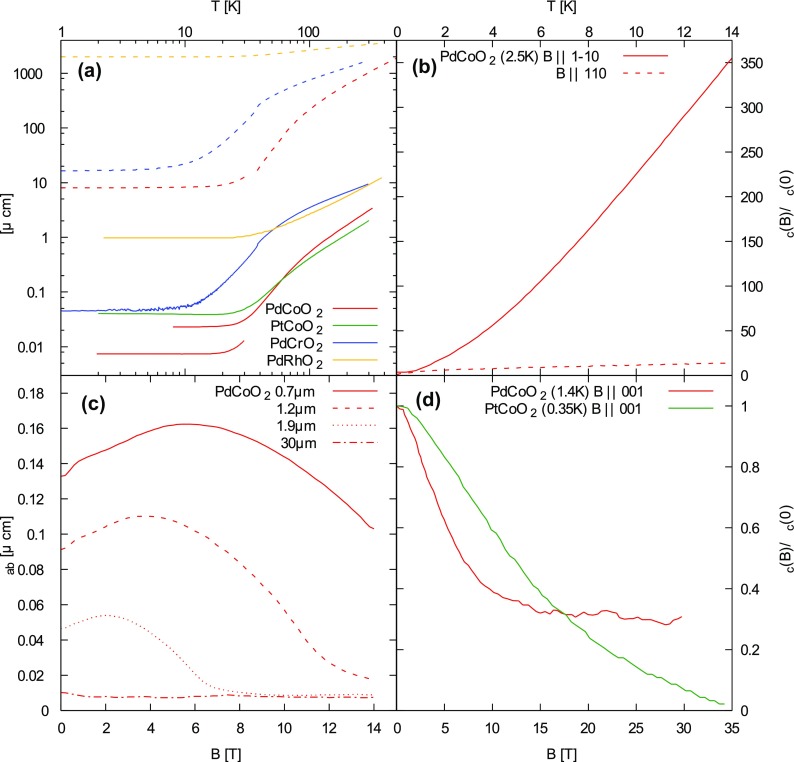
Resistivity of metallic delafossites (compiled from Refs. [41–43,49–52]). (a) The solid lines show the in-plane resistivity $\rho_{ab}$, while the dashed lines are out-of-plane resistivity $\rho_{c}$. $\rho_{ab}$ for two samples of $\;{\text{PdCoO}}_{2}$ of different residual resistivity are shown. At around 10 K both $\;{\text{PdCoO}}_{2}$ and $\;{\text{PtCoO}}_{2}$ have a shallow minimum in $\rho_{ab}$, and generally very similar temperature dependence and magnitude. The Néel transition in $\;{\text{PdCrO}}_{2}$ is seen as a small kink in both transport directions. (b) Transverse magnetoresistance $\rho_{c}\left(B\right)$ of $\;{\text{PdCoO}}_{2}$. When the magnetic field is aligned along the $\left[1\;\overset{¯}{1}\;0\right]$ direction, there is a huge response. (c) Channel width dependence of transverse magnetoresistivity $\rho_{ab}\left(B\right)$ in $\;{\text{PdCoO}}_{2}$ where the channel size is smaller than the mean free path. There is a complex dependence on magnetic field. (d) Negative longitudinal magnetoresistance $\rho_{c}\left(B\right)$ for $\;{\text{PdCoO}}_{2}$ and $\;{\text{PtCoO}}_{2}$.

The resistivity of $\;{\text{PdCoO}}_{2}$ has a super-linear temperature dependence not characteristic of the usual electron–phonon scattering mechanisms (Figure 4(a)). The presence of optical phonon modes with a characteristic temperature of 250K was suggested to account for this [49]. The form of the resistivity curve is similar in $\;{\text{PtCoO}}_{2}$ [41].

Meanwhile in the magnetic analogue $\;{\text{PdCrO}}_{2}$, the resistivity has a sub-linear temperature dependence, and is around three times higher than in $\;{\text{PdCoO}}_{2}$ [48]. This sub-linearity has been related to an extended range of magnetic scattering present related to the antiferromagnetic transition at $T_{N}=37$ K.

The low-temperature resistivity is of particular interest. In both $\;{\text{PdCoO}}_{2}$ and $\;{\text{PtCoO}}_{2}$ it falls to very low values, of the order of 0.01μΩcm. A slight upturn occurs below 20K, with a magnitude of about 5% of $\rho_{0}$ in $\;{\text{PtCoO}}_{2}$. This feature is so far unexplained. There is no sign of magnetic impurities that might contribute to a Kondo effect in these materials.

In $\;{\text{PdCoO}}_{2}$ the resistivity below 30 K can be fit to an exponentially activated form (notwithstanding the very small upturn) [43]. This is argued to be a consequence of the simple electronic structure. Only one band crosses the Fermi energy, and the resulting Fermi surface is a closed cylinder that does not touch the Brillouin zone boundary. There is therefore a minimum phonon wavevector required for an electron–phonon umklapp scattering event to occur. Such phonons have a characteristic temperature of 30 K, meaning that their population rises exponentially with this characteristic temperature. This leads to the observed exponentially activated resistivity. In the alkali metals, which also feature a single-band Fermi surface entirely contained within the first Brillouin zone, the equivalent temperature is only ∼4 K.

As a consequence of this suppression of electron–phonon umklapp scattering combined with low intrinsic disorder, the mean free path can be of the order of micrometers at low temperature. The result is the observation of channel size-dependent resistivity [51] (Figure 4(c)). Since umklapp scattering is one of the few mechanisms that generates resistivity in clean systems, its suppression means that the electron fluid at low temperatures has few ways to dissipate the momentum acquired under an applied electric field. When boundary scattering becomes the dominant source of relaxation, the result is the viscous flow of electrons. It has even been suggested that if conditions are right, it may be possible to observe phenomena such as second sound in these metals at low temperatures.

The out-of-plane resistivity remains metallic (continuously increasing with temperature) for $\;{\text{PdCoO}}_{2}$ [49], although the exponential activation at low temperatures is not present in this direction. This is as expected, as the Fermi surface touches the zone boundary in this direction and there are no forbidden electron–phonon interactions. The super-linear behavior at high temperature is also less pronounced than for the in-plane direction.

In $\;{\text{PdCrO}}_{2}$, the onset of antiferromagnetic order causes a small cusp in the resistivity as the Fermi surface reconstructs. Below $T_{N}$, the resistivity approximately follows $T^{3}$. This rapid power law arises from the suppression of scattering in the ordered state. Sub-linear temperature dependence is evident for both in-and -out-of-plane resistivity [48]. Extrapolation of the magnetic susceptibility points to a Curie-Weiss temperature $\Theta_{p}∼$500 K, an order higher than the Néel temperature. This implies a high degree of frustration. Specific heat [53] and neutron scattering [54] results point to the presence of magnetic fluctuations at temperatures between $T_{N}$ and $\Theta_{p}$. Magnon–electron scattering has been proposed to explain the elevated resistivity of $\;{\text{PdCrO}}_{2}$ compared to non-magnetic $\;{\text{PdCoO}}_{2}$.

#### 3.2.2. Magnetoresistance

Strong transverse magnetoresistance (MR) is a characteristic of these materials (Figure 4(b)), and becomes more pronounced at low temperatures. For in-plane current and magnetic field parallel to the c-axis, the orbital motion of quasi-particles around the cylindrical Fermi surface leads to MR of several hundred percent at low temperature. In absolute terms, however, this magnetoresistance is only of the order of a few μΩcm.

Striking results are obtained when the magnetic field is rotated in the plane and out-of-plane transport is recorded. In this case MR can be several thousand percent and depends sensitively on the in-plane field direction [55]. Sixfold patterns are observed that can be reproduced well using a semiclassical model of transport. Kohlers rule is also obeyed, implying that a single relaxation mechanism is dominant.

In the ordered state of $\;{\text{PdCrO}}_{2}$ the localized moments undergo a spin-flop for moderate magnetic fields applied in the plane [46]. A step is visible in the magnetoresistance curve at around 6.5 T. The same kind of spin-flop occurs at 5.3 T in semiconducting $\;{\text{CuCrO}}_{2}$, where the antiferromagnetic configuration is very similar. This emphasizes the local moment nature of the magnetism; while coupling to the conduction electrons in $\;{\text{PdCrO}}_{2}$ is significant enough to open a gap, the magnetic ordering of the $\;{\text{Cr}}^{3+}$ is largely unaffected by the presence of the conduction electrons.

Negative longitudinal magnetoresistance (LMR) has been observed in $\;{\text{PdCoO}}_{2}$ and $\;{\text{PtCoO}}_{2}$ (Figure 4(d)) at low temperatures and for particular field angles [52]. The occurrence of negative LMR in several materials has been taken as evidence that they belong to a class of topological materials, the Weyl semimetals. The negative LMR in those cases arises from the counterflow of surface currents in the presence of parallel electric and magnetic fields. A similar but more generic effect has been proposed that occurs in clean materials when a warped cylindrical Fermi surface undergoes Landau quantization in strong magnetic fields [56]. Points with quasi-linear dispersion appear where the Landau tubes intersect the Fermi energy, and these play the role of the Weyl points, driving the counterflow of surface currents.

A less exotic origin of negative magnetoresistance in clean materials is the suppression of boundary scattering by strong fields when the sample dimension approaches the electronic mean free path. Cyclotron orbits smaller than the sample size can increase the distance electrons travel between collisions with the boundary, thus causing a reduction in resistance. This condition is satisfied in $\;{\text{PdCoO}}_{2}$ at low temperatures for typical samples, but it is not clear if it can account for the angular dependence of the observed magnetoresistance.

#### 3.2.3. Hall effect

The Hall resistivity in $\;{\text{PdCoO}}_{2}$ and $\;{\text{PtCoO}}_{2}$ is nonlinear with magnetic field at intermediate temperatures, signaling the crossover from low- to high-field behavior [41,57]. Values of $\omega_{c}\tau$ can greatly exceed unity in magnetic fields accessible in the laboratory when aligned parallel to the c-axis. The temperature dependence of the Hall coefficient is generally monotonic, reflecting the single-band electronic structure, and the values reached in the high-field limit match well with the expectation of one electron per Pd/Pt ion.

**Figure 5.**
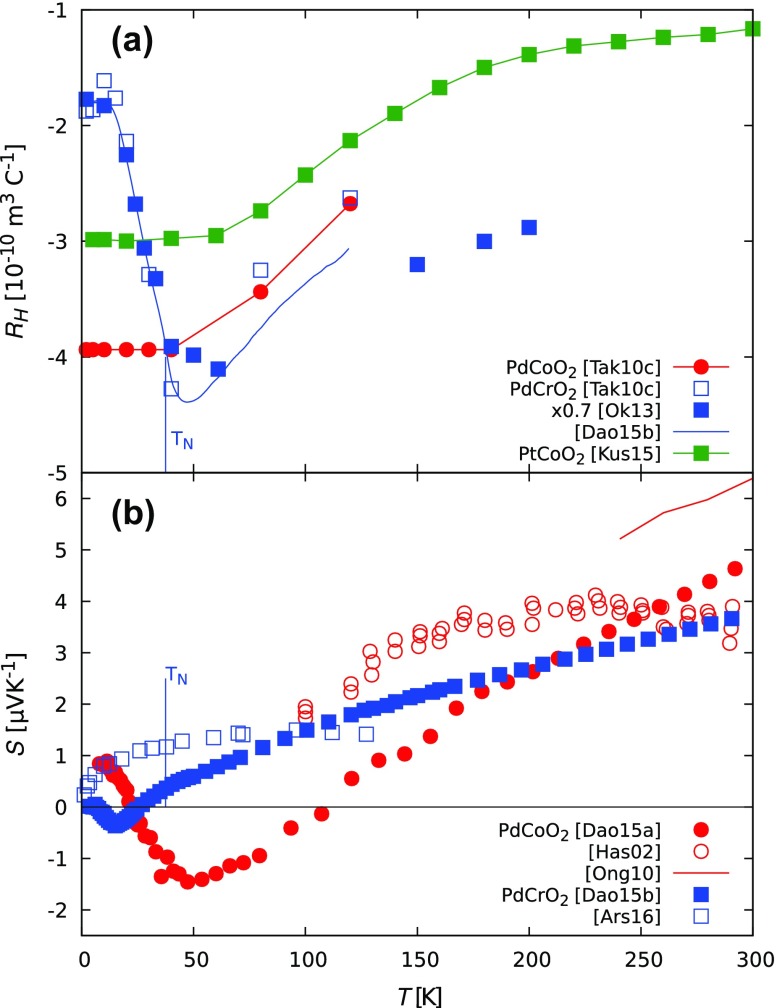
(a) Hall coefficient $R_{H}=\rho_{xy}/B$ of metallic delafossites in 8 T. Single-band $\;{\text{PdCoO}}_{2}$ [57] and $\;{\text{PtCoO}}_{2}$ [41] have a temperature-dependent Hall coefficient as the mean free path increases to beyond the cyclotron radius when the temperature is decreased, causing a crossover from weak-field to strong-field behavior. In contrast, $R_{H}$ in $\;{\text{PdCrO}}_{2}$ [45,57,58] has a complex temperature dependence due to the compensation between bands at low temperature as well as the presence of magnetic fluctuations around $T_{N}$. (b) Thermoelectric power of metallic delafossites in zero magnetic field. The thermoelectric power of $\;{\text{PdCoO}}_{2}$ single crystals [50] shows a phonon drag peak at low temperature and a linear dependence with temperature at high temperatures, as expected from calculations [59]. Older polycrystalline samples [25] may have been further from stoichiometry. There are also discrepancies between results on $\;{\text{PdCrO}}_{2}$ single crystals [58,61].

In $\;{\text{PdCrO}}_{2}$, with higher residual resistivity, $\omega_{c}\tau$-pagination reaches *O*(1). A more complex temperature and magnetic field profile surrounds the Néel transition. An early report interpreted this complexity as a sign of the unconventional anomalous Hall effect [57], as there was no obvious connection between the antiferromagnetic order parameter and magnitude of the Hall coefficient. A subsequent study showed that a two-band model incorporating magnetic breakdown could reasonably account for the nonlinearity of the Hall resistivity in the Néel state [45]. While the proposed breakdown fields are quite small, the antiferromagnetic gap opened in the Fermi surface is also found to be of the correct order of magnitude. A study of the temperature dependence of the Hall coefficient close to the Néel temperature revealed the impact of the short-range magnetic fluctuations on macroscopic transport [58]. Rather than being dominated by changes in diffuse scattering by magnetic excitations, the Hall coefficient smoothly interpolates between one-band and two-band electronic structures as a result of coherent scattering between hotspots by magnetic excitations. Hall effect data are shown in Figure 5(a).

#### 3.2.4. Thermoelectricity

$\;{\text{PdCoO}}_{2}$ and $\;{\text{PtCoO}}_{2}$ were suggested as interesting candidates for thermoelectric cooling on the basis of numerical calculations [59]. These showed that the c-axis Seebeck coefficient could be as large as $-200\;\mu \;\text{V}\;{\text{K}}^{-1}$, unusually large for a good metal. Combined with a limited electrical conductivity, the thermoelectric power factor $S^{2}/\rho$ could be large enough for applications to become feasible.

In the planar direction, the predicted sign is positive and the magnitude is much smaller, which was in accord with an early report on polycrystalline $\;{\text{PdCoO}}_{2}$ [25]. Owing to the tendency for single-crystal samples to grow as thin platelets, it has so far not been possible to measure the out-of-plane Seebeck coefficient, however, the measured in-plane values are in good agreement with the calculations [50]. The strong predicted anisotropy and sign change of the Seebeck coefficient with respect to crystalline direction are another unique aspect of these materials. In one suggestion, this sign difference could be exploited in a monolithic type of thermoelectric device [60]. Zero-field data for $\;{\text{PdCoO}}_{2}$ and $\;{\text{PdCrO}}_{2}$ are shown in Figure 5(b).

The temperature dependence of the in-plane Seebeck coefficient of $\;{\text{PdCoO}}_{2}$ is dominated by a linear component, as expected for a good metal. The slope of this component is close to the prediction of free electron theory [59], around $0.018\;\mu \;\text{V}\;{\text{K}}^{-2}$. At low temperature there is a pronounced negative peak.

This negative peak must be ascribed to phonon drag, whereby the flow of phonons along the thermal gradient exerts an additional pressure on the electrons. This effect is most pronounced at some fraction of the Debye temperature. The presence of phonon drag was confirmed by a study of the impact of sample purity on the size of the peak: cleaner samples yield a larger drag peak [50].

In the case of $\;{\text{PdCrO}}_{2}$ the experimental situation is not yet clear; two recent studies produce rather different results for the temperature dependence of the Seebeck coefficient. Ref. [58] shows a Seebeck coefficient dominated by a linear term with a weak negative peak at low temperature, much like $\;{\text{PdCoO}}_{2}$. The slope of the linear term is similar to that obtained in $\;{\text{PdCoO}}_{2}$, as expected because of the similarities in electronic structure. The low-temperature peak may arise from phonon drag or from a multi-band scenario as was used to explain the Hall effect data. In magnetic fields up to 8 T, the negative peak becomes more pronounced, which argues in favor of the multi-band scenario.

However, Ref. [61] reports a Seebeck coefficient with a rather different temperature dependence. It also shows a strong suppression under intense magnetic fields, which is not seen in the moderate applied fields used in Ref. [58]. The nonlinear form of the zero-field temperature dependence is somewhat unexpected, given the linear behavior in $\;{\text{PdCoO}}_{2}$, whose electronic structure should be very similar at high temperatures. The suppression of magnon drag with increasing magnetic field was invoked to explain the strong magnetic field dependence. It is difficult to reconcile the greatly different data reported in these two studies; both crystals appear to be of similar quality and exhibit the same Néel transition.

#### 3.2.5. Thermal conductivity

The anisotropy of the thermal conductivity of $\;{\text{PdCoO}}_{2}$ was extracted from a Montgomery-type experiment [50]. The results ruled out any possible application in energy conversion; the thermoelectric efficiency would be negligible because of a significant out-of-plane lattice thermal conductivity of around $50\;\;\text{W}\;{\text{K}}^{-1}\;{\text{m}}^{-1}$ at room temperature. The in-plane thermal conductivity is dominated by the electronic contribution and reaches ∼ $300\;\;\text{W}\;{\text{K}}^{-1}\;{\text{m}}^{-1}$ at 300 K, but with a Lorenz ratio approaching 1.1. This suggests that the lattice contribution to the thermal conductivity is much more significant than in the noble metals, for example, where the Lorenz ratio does not usually rise above unity.

#### 3.2.6. Nernst effect

The Nernst effect was studied across the antiferromagnetic transition in $\;{\text{PdCrO}}_{2}$ [58]. The Nernst effect is the thermoelectric equivalent of the Hall effect and is sensitive to changes in mobility and carrier number. In $\;{\text{PdCrO}}_{2}$, the Nernst coefficient also responds to the presence of the antiferromagnetic fluctuations that foreshadow long-range order. The response can be understood within the same framework as the Hall effect, where the opening of the gaps at the hotspots is smoothed by the presence of fluctuations. In the ordered two-band state, the Nernst coefficient is large and depends strongly on magnetic field, while in the paramagnetic single-band phase, it is small and of a magnitude compatible with a high carrier density. The crossover region is characterized by a sign change and a strong increase in magnitude.

#### 3.2.7. Discussion

The simplicity of the electronic structure in $\;{\text{PdCoO}}_{2}$ and $\;{\text{PtCoO}}_{2}$ leads to interesting transport properties even in the absence of strong correlations. These materials are self-organizing on the nanoscale, alternating conducting and insulating layers. In the case of $\;{\text{PdCrO}}_{2}$, the insulating layer also supports local moment magnetism. This unique material permits the study of symmetry breaking on transport properties without contaminating the conduction layer with magnetic ions.

## 4. Theoretical analysis

### 4.1. Electronic structure

Since its invention about five decades ago, density functional theory has witnessed a tremendous success story in predicting, explaining, and understanding the electronic properties of matter. This overwhelming progress was initiated by the fundamental work of Hohenberg, Kohn, and Sham, who established the electronic density as the key variable to access the properties of the ground state [62,63]. Nowadays, density functional theory is an integral and indispensable part of condensed matter research and materials science, which impressively complements experimental studies and has found its way into industrial laboratories [64,65].

Since calculations as based on density functional theory, usually called first principles calculations, do not need any input data other than the atomic numbers of the constituent atoms and their initial coordinates, they serve as an ideal and independent starting point for any investigation of materials. While summarizing recent first principles work, we largely follow the discussion presented in previous publications by our group, which should be consulted for a detailed account of all results [35,37,66,67]. In the present context, we mention only that all calculations were performed using the augmented spherical wave (ASW) method, which likewise is described in some detail elsewhere [68,69].

### ${\mathrm{PdCoO}}_{2}$ and ${\mathrm{PtCoO}}_{2}$

4.2.

A number of electronic structure investigations of these two Co-delafossites have been reported in the literature. Most of them focused on the extraordinary conductivity and specifically tried to clarify the composition of the wave functions at the Fermi energy. In particular, linear muffin-tin orbital calculations were performed by Seshadri et al. as well as by Okabe et al. [70,71]. The former authors, who also investigated ${\mathrm{PtCoO}}_{2}$, attributed the density of states at $E_{F}$ mainly to the Pd 4*d* states with only small contributions from the Co 3*d* and O 2*p* orbitals. In contrast, photoemission data were interpreted assuming the density of states at the Fermi energy arises exclusively from the Pd 4*d* states [17,18,72]. Furthermore, from the combination of photoemission spectroscopy and inverse photoemission spectroscopy it was concluded that the Fermi energy is located in a shallow minimum of the density of states and doping may thus cause rather high values of the thermoelectric power [25,72,73]. Hence, an investigation as that described below was needed to resolve the controversy by identifying the influence of the different atomic species and orbitals on the electronic properties and thereby to make closer connection with the photoemission data [66]. In contrast to previous calculations in the literature, which were based on the crystal structure data by Shannon, Rogers, and Prewitt [16], fully optimized lattice parameters and atomic positions as given in Table 1 were used.

**Table 1.** Experimental and calculated lattice parameters (in Å) and atomic positions.

Compound		a	c	$z_{O}$
${\mathrm{PdCoO}}_{2}$	exp.	2.8300	17.743	0.1112
	calc.	2.8767	17.7019	0.1100
${\mathrm{PtCoO}}_{2}$	exp.	2.8300	17.837	0.1140
	calc.	2.8989	17.458	0.1128

**Figure 6.**
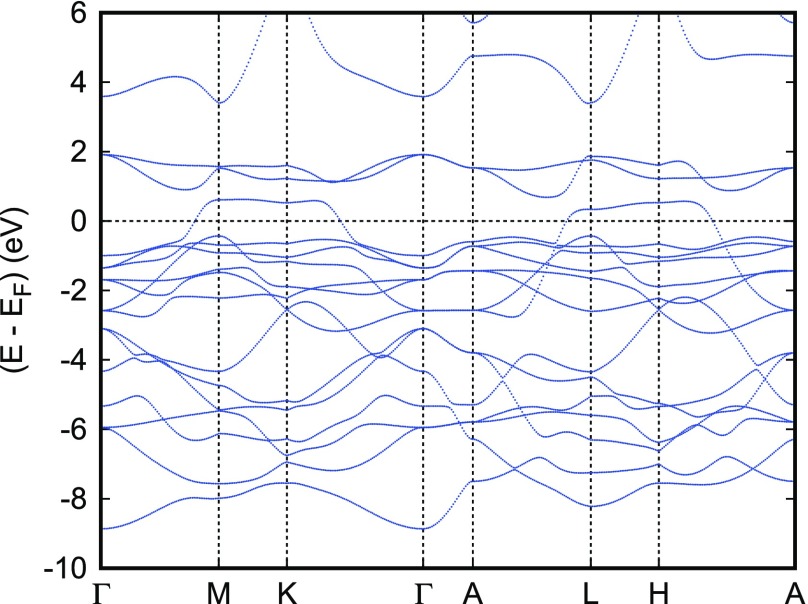
Electronic bands of $\;{\text{PdCoO}}_{2}$. Reprinted with permission from [66]. Copyright (2008) American Chemical Society.

**Figure 7.**
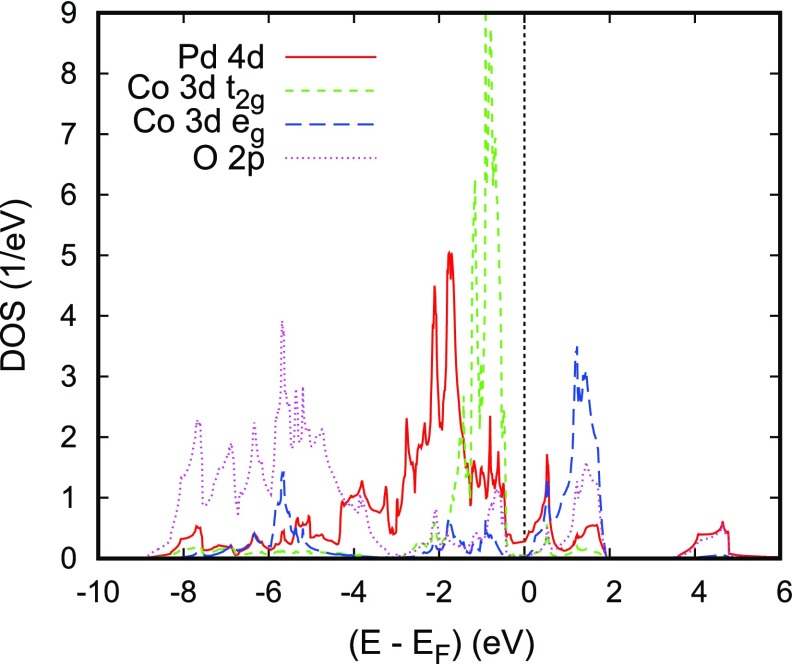
Partial densities of states (DOS) of $\;{\text{PdCoO}}_{2}$. Selection of Co 3*d* orbitals is relative to the local rotated reference frame, see text. Reprinted with permission from [66]. Copyright (2008) American ChemicalSociety.

In discussing the electronic properties of the two cobaltates, we start displaying the electronic bands along high-symmetry lines of the first Brillouin zone of the hexagonal lattice as well as the partial densities of states (DOS) in Figures 6 and 7, respectively. The somewhat complicated structure of both the electronic bands and the DOS results from the energetical overlap of the relevant orbitals in the energy interval shown. Yet, close to the Fermi energy this complexity clears up completely and only a single band straddles $E_{F}$, leading to a very simple Fermi surface. Since a detailed discussion of the results can be found in Ref. [66], we here focus on the most important findings. These include the crossover from predominant O 2*p* bands to a set of sharp peaks due to transition metal *d* states at about -4 eV and a clear separation of the Co 3*d* states into their $t_{2g}$ and $e_{g}$ manifolds due to the octahedral coordination by oxygen atoms. Note that the latter observation refers to a local rotated coordinate system with the Cartesian axes pointing approximately toward the oxygen atoms. Since the Fermi energy falls right between the $t_{2g}$ and $e_{g}$ manifolds, Co is found in a $d^{6}$ low-spin state. In this respect ${\mathrm{PdCoO}}_{2}$ is thus not unlike ${\mathrm{CuRhO}}_{2}$ to be considered below. However, while in the latter compound the *d* orbitals are fully occupied and, hence, allow for the semiconducting behavior, the missing electron in ${\mathrm{PdCoO}}_{2}$ leads to incomplete band filling and the finite conductivity. In conclusion, the above results confirm the picture of trivalent Co and monovalent Pd in a $d^{9}$ configuration [16–18]. At the same time, they clearly reveal the only tiny contribution of the Co and O states to the electrical conductivity, which is carried almost exclusively by the Pd 4*d* states.

**Figure 8.**
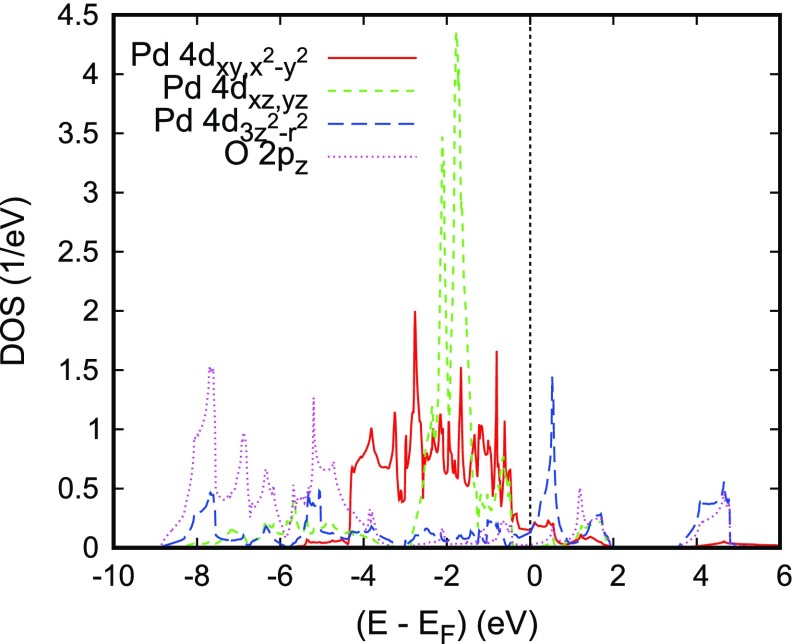
Partial Pd 4*d* and O $2p_{z}$ DOS of $\;{\text{PdCoO}}_{2}$. Reprinted with permission from [66]. Copyright (2008) American Chemical Society.

It is very instructive to further analyze the latter in terms of their five 4*d* partial DOS, which are shown in Figure 8. Since Pd is linearly coordinated by two oxygen atoms parallel to the *c* axis and has six Pd neighbors in the *a* - *b* plane, to this end the global coordinate system is used. With this choice, contributions from the $d_{xy}$ and $d_{x^{2}-y^{2}}$ as well as from the $d_{xz}$ and $d_{yz}$ states are identical. The Pd 4*d* partial DOS are strongly influenced by the linear coordination. Again, without going into details, we mention the strong σ-type $d_{3z^{2}-r^{2}}$-$p_{z}$ overlap along the *c* axis as indicated by the striking similarity of the corresponding partial DOS in the energy range from -9 to -4 eV as well as the broad Pd $d_{xy,x^{2}-y^{2}}$ bands reflecting the short in-plane Pd–Pd distances, which are very close to those of metallic Pd. Obviously, these latter states and the $d_{xy,x^{2}-y^{2}}$ states add the largest contributions to the total DOS at $E_{F}$, whereas that of the $d_{xz,yz}$ states is almost negligible. Finally, the sharp peak of the $d_{3z^{2}-r^{2}}$ partial DOS at about +0.6 eV can be clearly assigned to the almost dispersionless band along the lines M–K and L–H.

**Figure 9.**
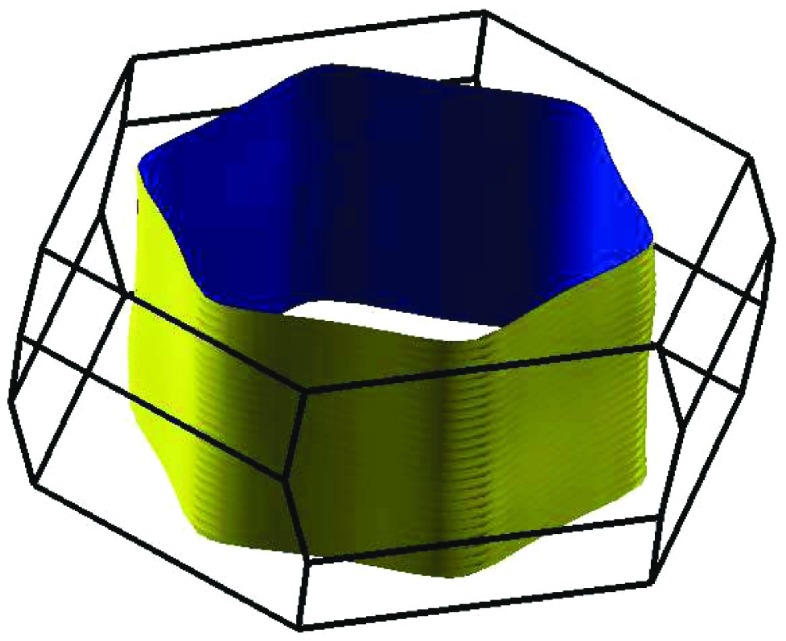
Fermi surface of $\;{\text{PdCoO}}_{2}$. Reprinted with permission from [66]. Copyright (2008) American Chemical Society.

Finally, the Fermi surface shown in Figure 9 underlines the strong quasi-two-dimensionality of the electronic states and, hence, explains the strong anisotropy in electrical conductivity.

The physical picture emerging from the above results agrees very well both with previous calculations [70,71] and more recent first principles studies [59,74,75]. They are also in agreement with photoemission and x-ray absorption data by Tanaka et al., Higuchi et al., and Noh et al. [17,44,72,76], who likewise attribute the metallic conductivity almost exclusively to the Pd 4*d* states and even regard $\;{\text{PdCoO}}_{2}$ as a metal–insulator stack structure [44]. These authors attribute the high conductivity to the strong dispersion of the conduction band, the large Fermi surface, and the long lifetime of the charge carriers. The extraordinary transport properties of $\;{\text{PdCoO}}_{2}$ were studied by Takatsu et al., by Ong et al., by Gruner et al., as well as by Daou et al., who reported on the strong anisotropy of the electrical conductivity [49,50] and the thermoelectric power [59,75]. This goes along with de Haas–van Alphen measurements of Hicks et al., who found anomalously low contributions from electron–phonon, electron–electron, and electron–impurity scattering to the resistivity [43]. Finally, the exceptional magnetoresistance has been discussed by Takatsu et al. as well as Kikugawa et al. [52,55].

There is a dispute about the detailed composition of the electronic wave functions at the Fermi surface. Arguments in favor of hybridized Pd $d_{3z^{2}-r^{2}}$- 5*s* orbitals were supported by Kimura et al. [77]. However, the above results demonstrate that the metallic conductivity is maintained by the in-plane $d_{xy}$ and $d_{x^{2}-y^{2}}$ orbitals and the in-plane part of the $d_{3z^{2}-r^{2}}$ orbitals to a similar degree with a somewhat greater influence of the former.

In passing, we mention very similar findings for ${\mathrm{PtCoO}}_{2}$. Yet, the anisotropy of the electrical conductivity is reduced in this compound due to the larger extent of the Pt 5*d* orbitals and the resulting increased overlap of the in-plane *d* states as well as with the O 2*p* states.

### ${\mathrm{CuFeO}}_{2}$

4.3.

As already mentioned before, one of the most attractive properties of the delafossites arises from the arrangement of transition metal ions on a 2D triangular lattice, which allows to study e.g. frustration effects, incommensurate and commensurate non-collinear magnetic order, unusual spin coupling, and multiferroic behavior. ${\mathrm{CuFeO}}_{2}$ and ${\mathrm{CuCrO}}_{2}$ have found much interest in this context and are therefore also considered in this overview.

The investigations of magnetic delafossite compounds has been from the very beginning accompanied by the search for distortions occurring at low temperatures in order to escape the geometric frustration. For this reason, the exact atomic and magnetic structure of many of these compounds has long been a matter of extensive dispute. This is not different for ${\mathrm{CuFeO}}_{2}$ [27,78]. Furthermore, there has been also some dispute about the possible occurrence of multiple magnetic phase transitions. From neutron diffraction data, Mekata et al. identified two magnetic transition for ${\mathrm{CuFeO}}_{2}$ at $T_{N1}=16$ K and $T_{N2}=11$ K. The magnetic phases required monoclinic and orthorhombic magnetic supercells of the undistorted rhombohedral unit cell with commensurate and incommensurate collinear arrangements of the localized $4.4\mu_{B}\;{\mathrm{Fe}}^{3+}$ moments [79–82]. More importantly, using x-ray and neutron diffraction measurements, Ye et al. indeed found structural distortions below 4 K accompanying the magnetic phase transitions and leading to a monoclinilattice with space group *C*2 / *m* [83]. In addition, analysis of spin-wave spectra gave strong hints at a 3D magnetic coupling [84]. It was also found that in response to a magnetic field the magnetic transition temperatures lower and an incommensurate structural distortion as well as ferroelectricity is induced [83]. Observation of a non-collinear–incommensurate phase in magnetic field was likewise taken as indicative of multiferroic behavior by Kimura et al. [82] and later on indeed identified in Al-doped ${\mathrm{CuFeO}}_{2}$ [85]. Further indication of multiferroic behavior was taken from inelastic neutron scattering data [84]. Ruttapanun et al. have pointed to the potential of Pt-doped ${\mathrm{CuFeO}}_{2}$ for thermoelectric applications [86].

Despite strong interest, only few electronic structure calculations for magnetic delafossite compounds had been reported [70,87–89]. This is possibly due to the still much debated atomic and magnetic structure. For that reason, only the ferromagnetic configuration was considered. Yet, the results were contradictory. Galakhov et al. reported on a ferromagnetic ground state for the rhombohedral $R\overset{¯}{3}m$ structure with a magnetic moment at the Fe site of about 0.9  $\mu_{B}$, much lower than the experimental value [87]. Furthermore, the Fe 3*d* $t_{2g}$ states were found above the Cu 3*d* states just at the Fermi energy, again in disagreement with both photoemission data and the fact that ${\mathrm{CuFeO}}_{2}$ is a semiconductor with an optical band gap of about 1.15 eV. In contrast, LDA+U calculations revealed a band gap of 2 eV and a magnetic moment of 3.76 $\mu_{B}$. However, the occupied Fe 3*d* states were located at about 9 eV below the valence band maximum and thus much too low [87]. More recently, Ong et al. found in their calculation a high-spin state with a magnetic moment of 3.78 $\mu_{B}$ per Fe and the Fe $3d\;t_{2g}$ spin-up states below the Cu 3*d* bands in agreement with photoemission and x-ray emission data [88]. However, again a finite optical band gap was arrived at only after taking into account local electronic correlations within the GGA+U scheme.

**Figure 10.**
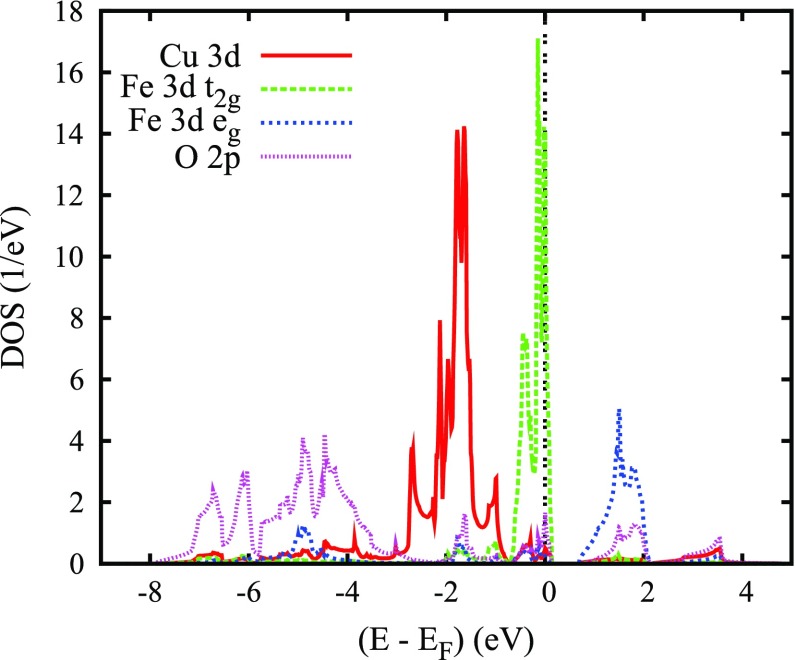
Partial densities of states (DOS) of rhombohedral $\;{\text{CuFeO}}_{2}$. Selection of Fe 3*d* orbitals in this and the subsequent figures is relative to the local rotated reference frame, see text. Reprinted with permission from [67]. Copyright (2008) American Physical Society.

We begin a detailed analysis by discussing the density of states (DOS) arising from spin-degenerate calculations for the rhombohedral structure as displayed in Figure 10. Crystal structure data by Ye et al. [83] are used throughout. As for ${\mathrm{PdCoO}}_{2}$, a crossover from dominating O 2*p* states to a sequence of sharp transition metal *d* states is observed at about -3 eV. While Cu is found in a monovalent $d^{10}$ configuration in good agreement with experiment, Fe assumes a $d^{5}$ state with the Fermi energy falling into the upper part of the $t_{2g}$ manifold. Note that in distinguishing the $t_{2g}$ and $e_{g}$ manifolds we again used the local rotated coordinate system with the Cartesian axes adjusted to an assumed perfect oxygen octahedron. Details can be found in Ref. [67].

In order to be in line with the previous work by Galakhov et al. as well as by Ong et al. [87,88], we next mention spin-polarized calculations for an assumed ferromagnetic state. Three different configurations were obtained corresponding to a low-spin, intermediate-spin, and high-spin moment located at the Fe site. The total energies as compared to the spin-degenerate configuration and the local magnetic moments are summarized in Table 2. All three ferromagnetic configurations are lower in energy than the spin-degenerate case with the high-spin state being the most stable as long as the lattice is restricted to be rhombohedral. The results thus confirm both the low-spin and high-spin calculations by Galakhov et al. as well as by Ong et al. In particular, they assign the differences between their findings to the existence of difference spin states.

In a second step, the monoclinic structure observed by Ye et al. is considered [83]. Since this monoclinic unit cell still contains one Fe atom it allows only for spin-degenerate and spin-polarized ferromagnetic calculations. The resulting total energies and magnetic moments are given in Table 2. While the latter are very similar to those obtained for the rhombohedral structure, the total energies are all lower by several mRyd with the largest energy lowering occurring for the high-spin state. In passing, we mention that the similarities between the results obtained for both structures extend also to the partial densities of states.

**Figure 11.**
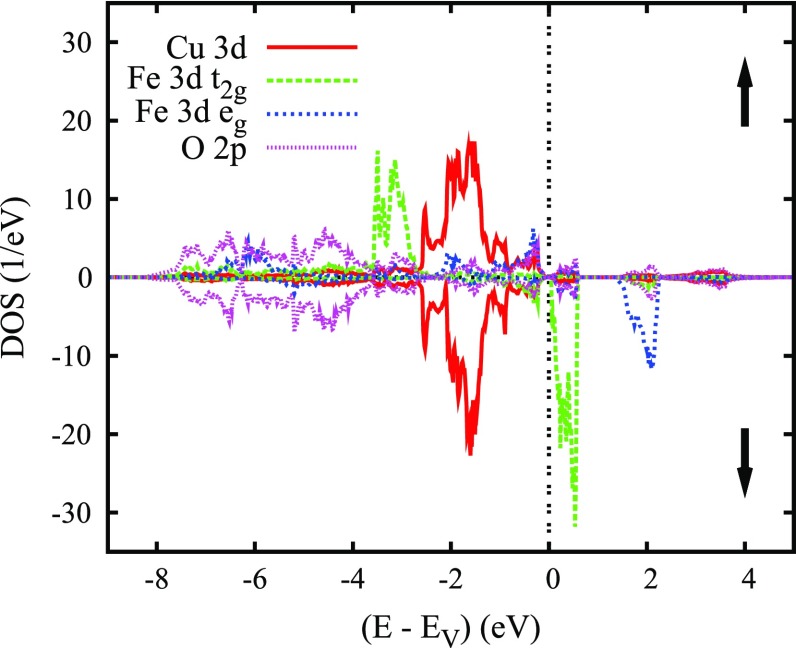
Partial densities of states (DOS) of monoclinic antiferromagnetic high-spin $\;{\text{CuFeO}}_{2}$. Reprinted with permission from [67]. Copyright (2008) American Physical Society.

Finally, we turn to calculations for the eightfold magnetic supercell proposed by Ye et al. [83]. The resulting partial DOS are displayed in Figure 11 and the local magnetic moments and total energy included in Table 2. Obviously, the antiferromagnetic state has the lowest energy as compared to all other configurations and Fe is found to be in a high-spin state in agreement with the neutron diffraction data by Mekata et al. [79,80]. Moreover, the calculation yields a band gap of 0.05 eV, in contrast to all previous results for ${\mathrm{CuFeO}}_{2}$. The results for the ferromagnetic high-spin states as well as the antiferromagnetic ground state have been nicely confirmed by Zhong et al., who, in addition, discussed the multiferroic behavior in terms of the hybridization of the Fe 3*d* and O 2*p* states [90]. In order to obtain a band gap in closer agreement with experiment, Hiraga et al. performed GGA+U calculations and obtained a band gap of about 1 eV for U=4 eV [91].

**Table 2.** Total energies (in mRyd per formula unit) and magnetic moments (in $\mu_{B}$) for different crystal structures and magnetic orderings of ${\;\text{CuFeO}}_{2}$.

Structure	Magn. order	ΔE	$m_{\mathrm{Fe}}$	$m_{O}$
rhombohedral	spin-deg.	0.0		
rhombohedral	ferro (LS)	-16.7	1.03	- 0.02
rhombohedral	ferro (IS)	-12.0	2.02	- 0.02
rhombohedral	ferro (HS)	-19.2	3.73	0.21
monoclinic	spin-deg.	-6.0		
monoclinic	ferro (LS)	-21.5	1.04	- 0.02
monoclinic	ferro (IS)	-19.0	2.08	- 0.02
monoclinic	ferro (HS)	-32.0	3.62	0.19
monoclinic	antiferro	-46.0	3.72	0.08

### ${\mathrm{CuCrO}}_{2}$

4.4.

${\mathrm{CuCrO}}_{2}$ is yet another example for strong geometric frustration effects coming with the predominantly antiferromagnetic coupling of rather well localized magnetic moments on a triangular lattice. However, unlike ${\mathrm{CuFeO}}_{2}$, ${\mathrm{CuCrO}}_{2}$ does not show any deformation of the atomic structure down to the lowest temperatures. The magnetic structure has been at the center of controversial debates for a long time. While early neutron powder diffraction work was in favor of an out-of-plane $120^{\circ }$ spin structure with a commensurate propagation vector along the $\left(\frac{1}{3},\frac{1}{3},0\right)$ direction [92], more recent studies revealed two possible structures, namely a helicoidal and a cycloidal structure with incommensurate propagation vector (0.329, 0.329, 0) below $T_{N}=24$ K [28]. More recently, single-crystal polarized neutron diffraction found the spiral plane to be parallel to the (110) plane [93]. Building on x-ray diffraction measurements, Kimura et al. reported on a slight deformation of the triangular lattice plane accompanying the magnetic ordering [94]. However, this finding has not been confirmed by other groups.

**Figure 12.**
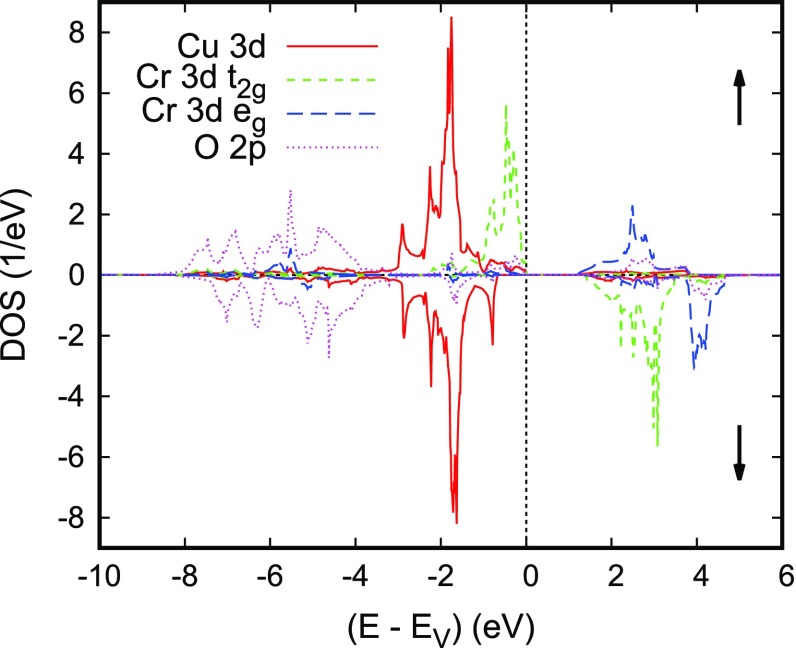
Partial densities of states (DOS) of rhombohedral ferromagnetic $\;{\text{CuCrO}}_{2}$. Reprinted from [112]. Copyright (2009) with permission from Elsevier.

According to neutron powder diffraction data, magnetic susceptibility measurements, electrical permittivity, and electrical polarization data of magnetically diluted ${\mathrm{CuCr}}_{1-x}M_{x}O_{2}$ with M=Al,Ga,Sc,Rh by Pachoud et al. the magnetic ground state turned out to be very robust against removal of Cr [95]. These authors thus concluded that the magnetic ground state of ${\mathrm{CuCrO}}_{2}$ is very different from that of ${\mathrm{CuFeO}}_{2}$.

In order to arrive at a better understanding of this material both spin-degenerate and spin-polarized calculations using the crystal structure data reported by Crottaz et al. [96] were performed. As for the other compounds discussed above, the partial densities of states as resulting from the spin-degenerate calculations have the lower part of the spectrum dominated by O 2*p* states, whereas the transition metal *d* states show rather sharp peaks above -4 eV. While Cu again is found in a monovalent $d^{10}$ configuration in close analogy with experimental findings, the Cr 3*d* states fall into half-occupied $t_{2g}$ and empty $e_{g}$ manifolds. From the $d^{3}$ configuration and the fact that $E_{F}$ is very close to the highest peak one would expect long-range ferromagnetic ordering of Cr moments of 3 $\mu_{B}$ in a spin-polarized calculation.

In view of the complex magnetic structure observed for ${\mathrm{CuCrO}}_{2}$ it is useful to start spin-polarized calculations by first considering an assumed ferromagnetic order. This procedure is well motivated by the previous work on ${\mathrm{CuFeO}}_{2}$, where we also started investigating an assumed ferromagnetic high-spin state before performing calculations for the antiferromagnetic ground state proposed by Ye et al. and found a strong similarity of the partial densities of states arising from these two configurations. Hence, we can learn a lot about the local electronic properties already from studying the ferromagnetic state.

Now, from spin-polarized calculations for an assumed ferromagnetic configuration of ${\mathrm{CuCrO}}_{2}$, a stable ferromagnetic configuration was obtained with magnetic moments of 3.0 $\mu_{B}$. Most importantly, the density of states as displayed in Figure 12 reveals a fundamental band gap of about 1.2 eV between the spin-up and spin-down Cr $3d\;t_{2g}$ states, which also carry the overwhelming part of the magnetic moment. Except for the lower filling of the Cr $3d\;t_{2g}$ states the partial densities of states look very similar to those of ${\mathrm{CuFeO}}_{2}$, which fact saves us a detailed discussion here.

**Figure 13.**
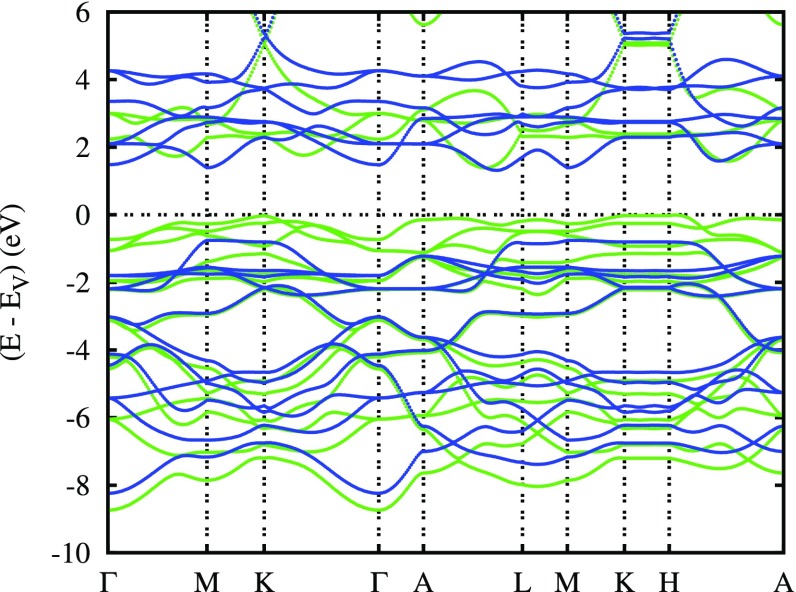
Electronic bands of rhombohedral ferromagnetic $\;{\text{CuCrO}}_{2}$. Green (blue) curves correspond to the majority (minority) spin bands. Reprinted from [112]. Copyright (2009) with permission from Elsevier.

The electronic bands along selected high-symmetry lines of the first Brillouin zone of the hexagonal lattice as displayed in Figure 13 reveal substantial 3D dispersion, which we attribute to the coupling between the layers. Yet, the dispersion is considerably reduced close to the valence band maximum. In addition, the highest occupied states at M and K are almost identical to those at L and H. Hence, there is almost no dispersion of these bands along the lines M–L and K–H. As a consequence, within a rigid band approximation, we would expect strongly localized bands arising from small hole doping, which would induce finite but still small Fermi velocities driving spin-dependent transport.

Performing GGA+U calculations for a variety of delafossites, Scanlon and co-workers considered an antiferromagnetic structure for ${\mathrm{CuCrO}}_{2}$ with ferromagnetic alignment within the sandwich planes and antiferromagnetic alignment of neighboring planes [97–99]. In addition, hybrid functional calculations were performed. While the authors also obtained a band gap separating occupied *d* states from empty Cr $3d\;e_{g}$ bands of about 1, 2, and 3 eV from their GGA, GGA+U, and hybrid functional calculations, respectively, their results are at some variance with those presented above. In particular, Cu 3*d* and Cr $3d\;t_{2g}$ states are both found in the same energy interval ranging from about -3 eV to the valence band maximum and bands close to the latter mainly trace back to the Cu states [97–99]. A very similar order of bands was obtained by Hiraga et al. [91].

**Figure 14.**
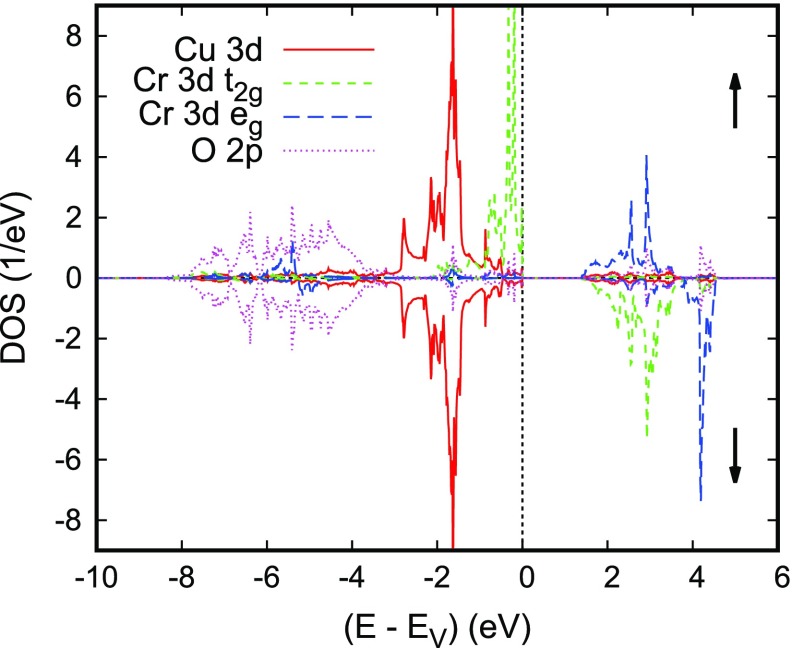
Partial densities of states (DOS) of hexagonal antiferromagnetic $\;{\text{CuCrO}}_{2}$.

**Figure 15.**
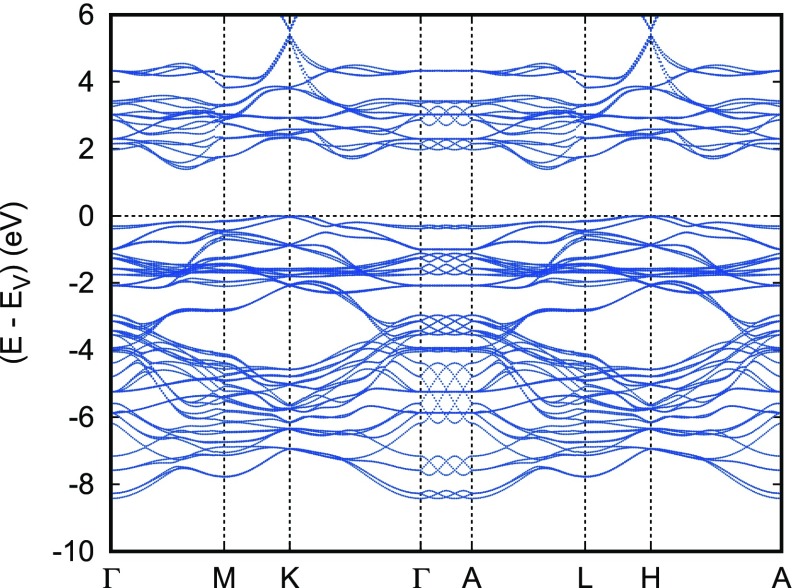
Electronic bands of hexagonal antiferromagnetic $\;{\text{CuCrO}}_{2}$.

In order to arrive at a more complete picture, we complemented the above calculations for the assumed ferromagnetic state with calculations for the antiferromagnetic structure proposed by Scanlon and co-workers. This structure requires a hexagonal rather than rhombohedral unit cell with a doubling of the hexagonal *c* axis and it comprises six formula units. The resulting partial densities of states and electronic bands are displayed in Figures 14 and 15. Note that while representing the electronic band structure we used the Brillouin zone of the original small cell and, hence, obtain threefold band oscillations along the line Γ-A. Apart from that, both the band structure and the partial densities of states are very similar to those obtained for the assumed ferromagnetic order and thus confirmed our above expectations, namely that the electronic properties are already very well described by those for an assumed ferromagnetic state. Still, especially the spin-majority Cr $3d\;t_{2g}$ bands at the valence band maximum are much more localized in the antiferromagnetic configuration. Nevertheless, the discrepancy as compared to the results by Scanlon et al. are not yet resolved. As a consequence, the predominant character of the wave function occurring on small hole doping is not known.

The issue has been critically discussed from both an experimental and a theoretical point of view by Yokobori et al., who combined photoemission spectroscopy, soft x-ray absorption spectroscopy, and electronic structure calculation within the LDA+U-pagination approximation as applied to the rhombohedral ferromagnetic configuration [100]. These authors confirmed the above results with the Cr $3d\;t_{2g}$ dominating the valence band maximum and the fully occupied Cu 3*d* bands found well below. Nevertheless, their x-ray absorption spectra on ${\mathrm{CuCr}}_{1-x}{\mathrm{Mg}}_{x}O_{2}$ showed a strong sensitivity to the Mg content indicating the hole will be doped into the Cu sites in contradiction to their photoemission spectra. In order to resolve the issue, Yokobori et al. proposed strong Cu 4*s* -Cr 3*d* charge transfer via the O 2*p* states. Nevertheless, this puzzling situation is still awaiting further insight.

Calculations of Jiang et al., which took into account non-collinear spin arrangements, revealed predominantly in-plane exchange interactions and an incommensurate spin–spiral structure with a (110) spiral plane and a screw-rotation angle close to $120^{\circ }$ in agreement with experimental data [93,101]. While spin–orbit interaction was shown to have only minor influence on the electronic properties spin frustration had a stronger effect on the *d* – *p* hybridization. Finally, Monte Carlo simulations using exchange parameters extracted from supercell calculations led to a Néel temperature of 29.9 K, again in acceptable agreement with the experimental findings [101]. Recent Monte Carlo simulations taking small lattice distortions into account yield a Néel temperature of $T_{N}≃27\;K$. Furthermore, a connection between the emergence of spin helicity below $T_{N}$ and ferroelectricity could be established [29].

### ${\mathrm{CuRhO}}_{2}$

4.5.

Finally, we turn to yet another delafossite compound, which has recently attracted much interest from a completely different perspective, namely as a very promising candidate for applications in thermoelectricity and water splitting [33,37,102,105]. Kuriyama et al. and Shibasaki et al. observed a room temperature thermopower of 130 $\mu VK^{-1}$ and of 70 $\mu {VK}^{-1}$, respectively, for ${\mathrm{CuRh}}_{0.9}{\mathrm{Mg}}_{0.1}O_{2}$ [33,102]. In addition, the former authors reported a figure of merit ZT≈0.15 at 1000 K [33].

There were reports on the use of ${\mathrm{CuRhO}}_{2}$ as a photocathode for water splitting under visible light [104,105]. Application in this field benefits from the fact that the band edges are optimally adapted to the water oxidation and reduction redox potentials [104]. Gu et al. attributed the stability of the delafossites as photocathodes to the fact that the electron acceptor levels are strongly dominated by the B-type atoms, i.e. the Rh *d* levels and pointed to the high sensitivity of the stability and efficiency to even small Cu 3*d* contributions in this energy range [104]. Thus, there was and still is a very high motivation to understand the electronic properties of ${\mathrm{CuRhO}}_{2}$ in detail.

Our calculations were based on the crystal structure data by Oswald et al. [106], who determined the lattice constants as a=3.08 Å and c=17.09 Å. Since these authors did not measure the internal oxygen parameter, we performed a structural optimization leading to a value of $z_{O}=0.10717$, which was used in all subsequent calculations [37].

**Figure 16.**
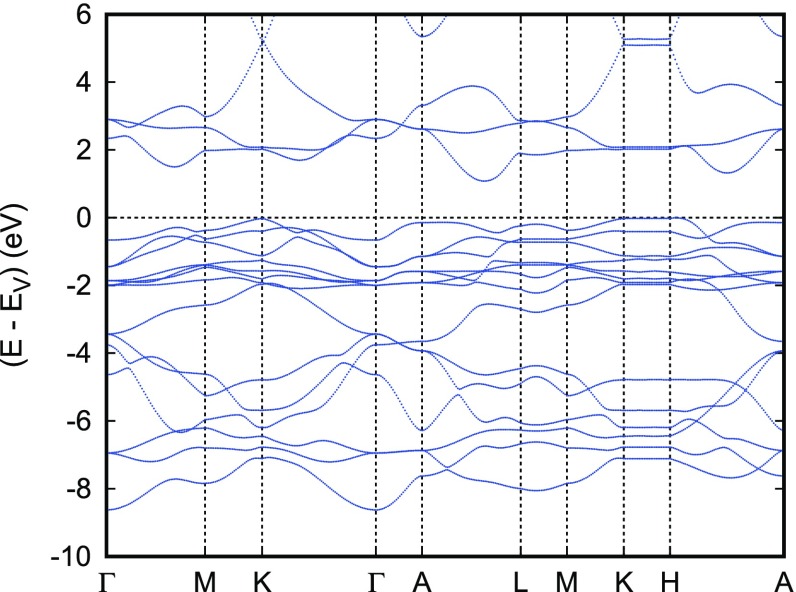
Electronic bands of $\;{\text{CuRhO}}_{2}$. Reprinted with permission from [37]. Copyright (2009) American Physical Society.

**Figure 17.**
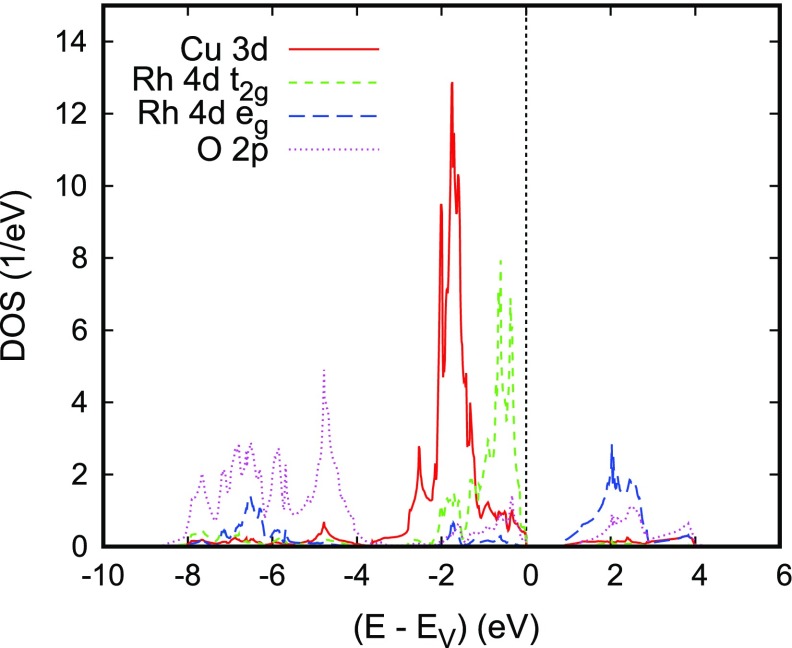
Partial densities of states (DOS) of $\;{\text{CuRhO}}_{2}$. Selection of the Rh 4*d* orbitals is relative to the local rotated reference frame, see text. Reprinted with permission from [37]. Copyright (2009) American Physical Society.

The electronic bands along selected high-symmetry lines of the first Brillouin zone of the hexagonal lattice and the partial densities of states (DOS) are displayed in Figures 16 and 17, respectively. Again, we observe the well-known order of states with the crossover from O 2*p* -dominated bands to sharp transition metal peaks at about -4 eV. As for the other Cu-based delafossites discussed above, Cu is found in a monovalent $d^{10}$ configuration in close analogy with the experimental findings with the major 3*d* peaks well below the valence band edge. The Rh 4*d* states clearly exhibit splitting into a fully occupied $t_{2g}$ manifold and empty $e_{g}$ bands, which are separated by the fundamental band gap. The calculated value of the latter is about 0.85 eV, which is somewhat smaller than the experimental value of 1.9 eV. Rh is thus found in a $d^{6}$ configuration. The situations is not unlike that of ${\mathrm{CuCrO}}_{2}$, where, however, only the spin majority $t_{2g}$ states are occupied and the spin minority $t_{2g}$ bands are shifted upward to join the $e_{g}$ bands above the band gap.

It is interesting to note the band finite dispersion parallel to Γ-A indicative of considerable three-dimensionality arising from the coupling between the layers. This is contrasted with the barely noticeable dispersion particularly along the line K–H. Yet, both the finite dispersion along Γ-A and the flat bands along K–H have been also observed for the other delafossite materials.

**Figure 18.**
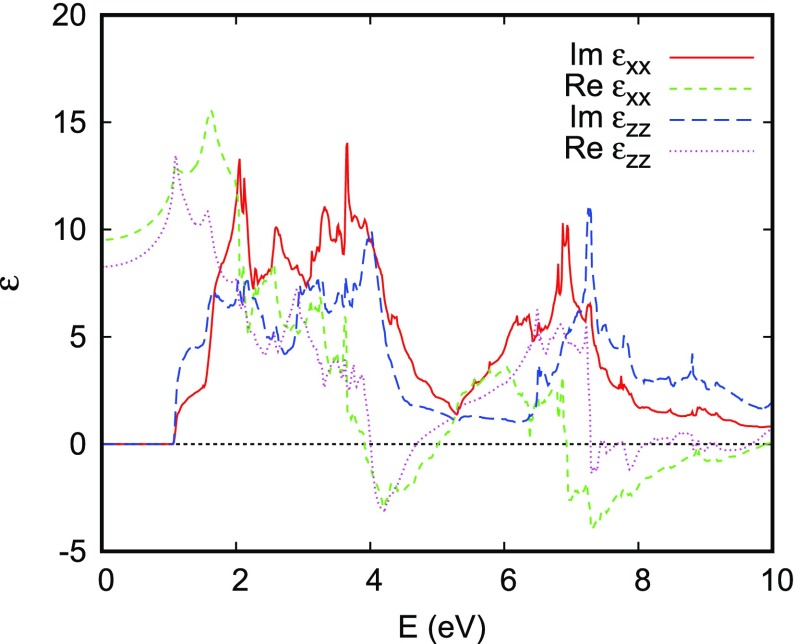
Dielectric function of $\;{\text{CuRhO}}_{2}$.

Further insight about the electronic properties is obtained from the optical spectra. The real and imaginary parts of the dielectric function as calculated within linear-response (see Ref. [69] for more details) are shown in Figure 18. Obviously, the asymmetry between the in-plane and out-of-plane directions is not reflected in the absorption gap following from the imaginary part of the dielectric function. In fact the gap is very close to 0.85 eV in all three directions, which is most likely to exceed the Hund’s rule coupling. Therefore, the low-spin $4d^{6}$ configuration of ${\mathrm{Rh}}^{3+}$ is expected to be the ground state. This is consistent with earlier findings by Singh for ${\mathrm{CuCoO}}_{2}$, where the Co ions adopt the low-spin $3d^{6}$ configuration [89].

**Figure 19.**
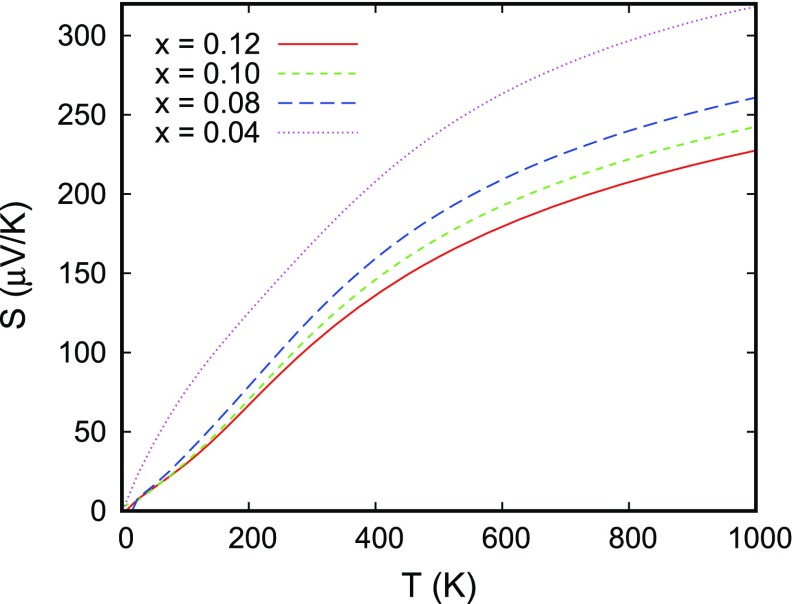
Thermopower *S* of $\;{\text{CuRhO}}_{2}$ for different hole doping levels. The curves display the average of all three diagonal components of the tensor.

Finally, we discuss the thermoelectric properties as evaluated from Boltzmann theory [69,107]. The transport properties are usually expressed in terms of the Onsager transport coefficients (see also Ref. [37] for details). The calculated Seebeck coefficients for different hole doping levels are displayed in Figure 19. Note that the curves show the average of all components of the diagonal of the respective tensor. As expected, the thermopower strongly decreases with increased hole doping. In addition, it shows the characteristic bending toward smaller increase at high temperatures. As concerns especially the shape of these curves, the results are in almost perfect agreement with the experimental data as shown in Figure 3 of our own group, the calculations of Usui et al. [103], who likewise used the Boltzmann approach, and with the experimental data by Kuriyama et al. [33]. Nevertheless, the absolute scaling of the thermopower is at variance both between the calculations and between different experiments. This should be related to the fact that calculated Seebeck coefficients are rather sensitive to details of the crystal structure. In addition, in applying Boltzmann theory, all calculations performed so far used a rigid band picture in order to mimic doping effects. Yet, a doping level of 0.1 holes per formula unit is not a small perturbation. For this reason, ${\mathrm{CuRh}}_{0.9}{\mathrm{Mg}}_{0.1}O_{2}$ should be regarded a metal rather than a doped semiconductor as is indeed done in the literature. Nevertheless, in view of these limitations, the above results are remarkably convincing.

## 5. Conclusions and perspectives

It is remarkable that going from $d^{9}$ to $d^{10}$ monovalent cations at the A site of $\;{\text{A}}^{+}\;{\text{M}}^{3+}\;{\text{O}}_{2}$ delafossite the electronic ground state could switch from ‘ultra’ metallicity as in $\;{\text{PdCoO}}_{2}$ to semiconductors for $\;{\text{CuCrO}}_{2}$ or $\;{\text{CuRhO}}_{2}$. Accordingly, very different physics in this class of materials can be studied as illustrated by a few examples leading, for instance, to multiferroicity when A is a $d^{10}$ monovalent cation in the case of $\;{\text{CuFeO}}_{2}$ and $\;{\text{CuCrO}}_{2}$. Their triangular lattices of localized magnetic moments at the M site is responsible for magnetic frustration which is lifted by antiferromagnetic ordering at rather low Néel temperatures of $T_{N}=16\;K$ and $T_{N}=24\;K$ for $\;{\text{CuFeO}}_{2}$ and $\;{\text{CuCrO}}_{2}$, respectively. For both compounds, the electronic spin-polarized structure calculations are consistent with the experimental observations that the antiferromagnetic semiconductors states are the most stable with high-spin states for $\;{\text{Fe}}^{3+}$ or $\;{\text{Cr}}^{3+}$. These calculations also reveal energy gaps at $E_{F}$ fitting with the poor conducting behavior. However, the limit of these calculations lies in the prediction of the magnetic structure for which subtle changes in the in-plane and out-of-plane exchange energies lead to very different antiferromagnetic structures below $T_{N}$ as the 4SL collinear structure in $\;{\text{CuFeO}}_{2}$ and the incommensurate structures, helicoidal or cycloidal in $\;{\text{CuCrO}}_{2}$. Another challenge for the calculations is created by the prediction of the thermoelectric properties. In these $d^{10}$ delafossites, substituting $\;{\text{Mg}}^{2+}$ for $\;{\text{Cr}}^{3+}$ or $\;{\text{Rh}}^{3+}$, leads to interesting thermoelectric properties characteristic of p-type materials which can be described as doped semiconductors in the case of $\;{\text{CuCr}}_{1-x}\;{\text{Mg}}_{x}\;{\text{O}}_{2}$ but as metals in the case of heavily doped $\;{\text{CuRh}}_{1-x}\;{\text{Mg}}_{x}\;{\text{O}}_{2}$ (x≃0.1).

Let us also remark that the band structure of delafossites entails Dirac cones, especially the one of $\;{\text{CuRhO}}_{2}$ [37]. Since they are far away from the Fermi energy they do not seem to have much influence on the physical properties. Yet, the Dirac cones could be brought closer to the Fermi energy in a capacitor geometry through the use of gate biases, see e.g. [108,109], to provide us with the first oxide topological insulator.

The large splitting of the 3d (or 4d) $t_{2g}$ and $e_{g}$ orbitals at $E_{F}$ in $\;{\text{CuCrO}}_{2}$ (or $\;{\text{CuRhO}}_{2}$) which accounts for the physical properties of these delafossites is in marked contrast with the incomplete band filling of $d^{9}\;\;{\text{Pd}}^{+}$ in $\;{\text{PdCoO}}_{2}$ or $\;{\text{PdCrO}}_{2}$ with Pd 4d states almost exclusively responsible for electrical conductivity. More particularly, the $d_{xy,x^{2}-y^{2}}$ orbitals add the largest contribution to the DOS at $E_{F}$. It results a simple Fermi surface, characteristic of quasi-2D electronic states. Thus, $\;{\text{PdCoO}}_{2}$ can be regarded as a textbook example of quasi-2D metals, which are also of high interest as paradigmatic candidates for angle-resolved photoemission spectroscopy line shape studies [110]. The stacking of Pd conducting layers alternating with a quasi-insulating $\;{\text{CoO}}_{2}$ layers leads to effects rarely seen in condensed matter. Although the 2D character is reflected in the anisotropic resistivities, the thermal conductivity κ being dominated by the lattice contribution, κ is much less anisotropic. As a result, $\;{\text{PdCoO}}_{2}$ is a metal with anomalously large κ values as compared to the values reported for noble metals. The thermoelectric power follows the prediction of the free-electron theory and, for $\;{\text{PdCrO}}_{2}$, where the ‘insulating layer’ contains a paramagnetic cations ($\;{\text{Cr}}^{3+}$; S=3/2), the physics becomes more complex. For instance, the Nernst coefficient is found to become large below $T_{N}$ with a very large sensitivity to the magnetic field. It must be also emphasized that these metallic delafossites belong also to the Weyl semimetals, a class of topological materials. This hypothesis has been put forward to explain the negative longitudinal magnetoresistance. Finally, growth of thicker crystals is needed for testing the prediction of high Seebeck coefficient along the transverse direction (S≃ $200\;\mu \;\text{V}\;{\text{K}}^{-1}$ along the c axis) together with a moderate out-of-plane resistivity. However, the κ measurement revealing very large values even for the transverse direction are not in favor of any applicability of these material in thermoelectricity-based waste-heat recovery.

Following the graphene original physics, the delafossites compounds, which 2D structure can be described as a natural 1:1 epitaxy of a metal $d^{9}$ or insulator $d^{10}$ metal layers with a $\;{\text{MO}}_{2}$ layer of the $\;{\text{CdI}}_{2}$-type, which can be diamagnetic or paramagnetic at room temperature with possibly an antiferromagnetic order at low *T*, offer a broad range of combinations to generate new physical properties. In that respect, there remains room for the chemists to produce materials of original compositions, to grow larger crystals to characterize all anisotropic properties – including the thermopower and thermal conductivity – and for the physicist, beyond all measurements still to be done, to improve the modeling of these properties.

Finally, it must be emphasized that the selected examples described in this review paper represent only a few compounds among a very broad family of delafossites. Looking beyond the oxides at compounds crystallizing in similar structures, it must be also mentioned that three exists many layered $\;{\text{AMX}}_{2}$ compounds for other chalcogen ions (X=S, Se, Te) for which the physics is also very exciting as complex antiferromagnetism in $\;{\text{AgCrS}}_{2}$ and $\;{\text{AgCrSe}}_{2}$ or the photovoltaic effects in CIGS ($\;{\text{CuGa}}_{1-x}\;{\text{In}}_{x}\;{\text{Se}}_{2}$). However, to the best of our knowledge, delafossite-like sulfides or selenides with metallicity as good as that of $\;{\text{PdCoO}}_{2}$ keep on escaping synthesis. Again, this outlines the importance of oxides to generate unusual properties.
